# Aspirin enhances endometrial decidualization markers *in vitro* among women with and without endometriosis

**DOI:** 10.1530/RAF-25-0034

**Published:** 2026-03-26

**Authors:** Edward R McClellan, Xiangying Xue, Prodyot K Chatterjee, Nathaniel Hyman, Rachel A Bennett, Randi H Goldman, Peter K Gregersen, Christine N Metz

**Affiliations:** ^1^ Northwell, New Hyde Park, New York, USA; ^2^ Department of Obstetrics and Gynecology, Division of Human Reproduction, Manhasset, New York, USA; ^3^ Zucker School of Medicine, Uniondale, New York, USA; ^4^ AMEDD Student Detachment, 187th Medical Battalion, 32nd Medical Brigade, Fort Sam Houston, Texas, USA; ^5^ Feinstein Institutes for Medical Research, Northwell Health, Manhasset, New York, USA

**Keywords:** aspirin, decidualization, endometrial stromal cells, endometriosis, proliferation

## Abstract

**Graphical Abstract:**

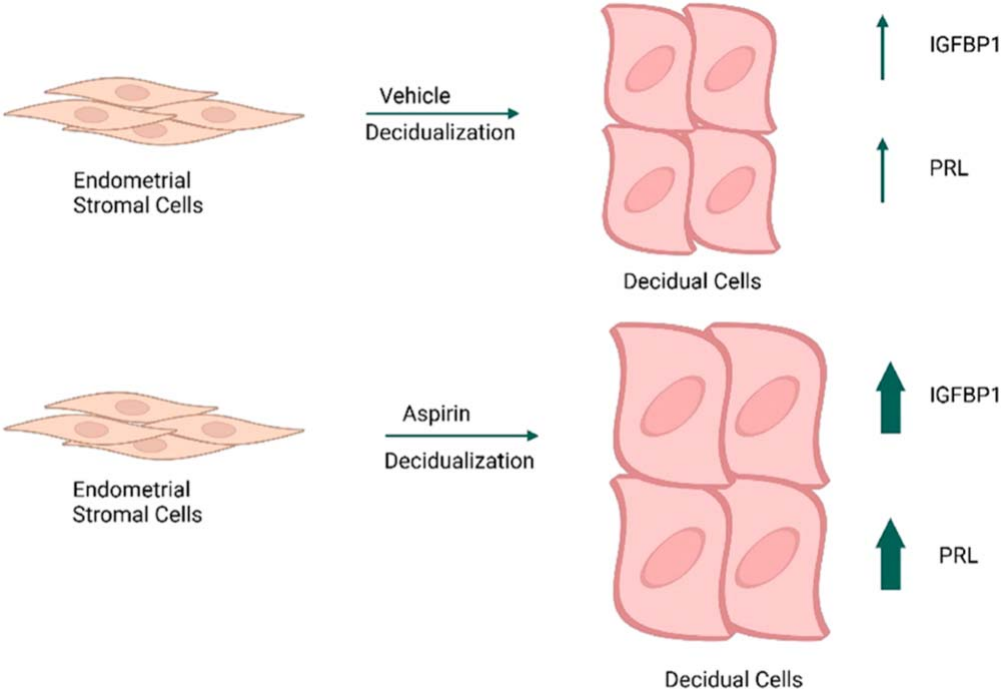

**Abstract:**

Decidualization of human endometrial stromal cells (ESCs) precedes the successful implantation of a human embryo. Improper decidualization has been reported in numerous conditions associated with infertility, including endometriosis. Multiple pathways, including aberrant cyclooxygenase (COX) expression, are associated with improper decidualization in endometriosis. Still, prior studies have not investigated the impact of aspirin (ASA) on ESC decidualization. After pre-treatment with vehicle or ASA (1–2.5 mM), ESCs were treated with 8-bromoadenosine 3′,5′-cyclic monophosphate sodium salt (cAMP) or cAMP + medroxyprogesterone acetate (MPA) to stimulate decidualization markers. Insulin-like growth factor-binding protein-1 (IGFBP1) and prolactin (PRL), biomarkers of decidualization, were measured in culture supernatants by ELISA (*n* = 12). Proliferation assays were performed (*n* = 10), and cytotoxicity was assessed using neutral red staining (*n* = 10). Results were converted to fold-change with vehicle-control = 1. The potential mechanism of action was investigated using western blotting for AKT phosphorylation. ASA (1 and 2.5 mM) pre-treatment increased decidualization markers (IGFBP1 and PRL) compared to vehicle treatment by both cAMP- and cAMP + MPA-treated ESCs without inducing significant cytotoxicity. Data suggest that ASA (2.5 mM) inhibits AKT phosphorylation to promote decidualization. While ASA (1 mM) did not significantly affect ESC proliferation, ASA (2.5 mM) significantly reduced proliferation compared to vehicle treatment. For all outcome measures, there were no statistically significant differences in the effects of ASA on ESCs obtained from women with vs without endometriosis.

**Lay summary:**

The human uterus prepares for pregnancy through natural changes to the lining of the uterus. In this process, the cells that line the inside of the uterus change to support implantation of a fertilized egg and growth of an embryo. Infertility and endometriosis, a condition where uterine-like cells grow outside of the uterus (e.g. ovaries, intestines, and other organs in the female pelvis), have been associated with a failure of these natural changes to the lining of the uterus. Prior studies have mostly relied on invasive procedures, such as uterine biopsies, to study these cells. Instead of painful uterine biopsies, we used period blood (or menstrual effluent, which contains shed uterine lining) to isolate and study the cells that line the inside of the uterus. We discovered that aspirin improves the ability of these cells to undergo the natural changes that prepare for pregnancy, without causing harm to the cells. These laboratory-based findings pave the way for future research to study the effects of aspirin on the cells that line the uterus among women with infertility and endometriosis who are taking aspirin.

## Introduction

Decidualization of endometrial stromal cells (ESCs) is an essential, well-regulated differentiation process that precedes the successful implantation of a human embryo (Gellersen & Brosens 2014, Okada *et al.* 2018). Improper decidualization has been reported in infertility and recurrent pregnancy loss (Teklenburg *et al.* 2010, Gellersen & Brosens 2014, Okada *et al.* 2018, Dambaeva *et al.* 2020), endometriosis (Barragan *et al.* 2016, Warren *et al.* 2018, Nayyar *et al.* 2020), and preeclampsia (Garrido-Gomez *et al.* 2017). Reduced decidualization at the implantation site predisposes to early pregnancy failure (Muter *et al.* 2021). In 2023, the European Society of Human Reproduction and Embryology prioritized research into decidualization as a therapeutic target for implantation failure (ESHRE Working Group on Recurrent Implantation Failure 2023). Endometriosis is associated with both infertility and defective endometrial decidualization (Barragan *et al.* 2016, Warren *et al.* 2018, Nayyar *et al.* 2020, Liao *et al.* 2024).

Multiple regulatory pathways have been implicated in the impaired decidualization response observed in endometriosis (Cai *et al.* 2022). Loss of AKT phosphorylation is an essential aspect of ESC decidualization (Fabi *et al.* 2017). Higher AKT phosphorylation has been reported in human ESCs obtained from women with endometriosis compared to unaffected controls (Cinar *et al.* 2009). Increased AKT phosphorylation in the setting of endometriosis has been implicated in endometrial dysregulation (Lee & Kim 2014). Aberrant endometrial COX-2 expression has also been reported in endometriosis (Teague *et al.* 2010). COX-1 and COX-2 have both been implicated in proper decidualization with COX-1 levels decreasing during decidualization (St-Louis *et al.* 2010).

Aspirin (ASA), an inhibitor of COX-1 and COX-2, is safe when taken prior to conception and may increase implantation and live birth in certain patients (Connell *et al.* 2017, Naimi *et al.* 2021). However, administration of ASA in assisted reproduction remains controversial (Siristatidis *et al.* 2016, Wang *et al.* 2017, Glujovsky *et al.* 2020). It is hypothesized that ASA improves uterine and ovarian blood flow (Rubinstein *et al.* 1999) and inhibits thromboxane synthesis by inhibiting the COX-1 pathway (Walsh & Strauss 2021, Boelig *et al.* 2024), thereby preventing thrombosis in placental vasculature (Fishman *et al.* 1995) and reducing placental vasculopathy (Boelig *et al.* 2024). COX-1 inhibitors can also lead to loss of AKT phosphorylation (Altintop *et al.* 2023).

ESC differentiation into decidual stromal cells that secrete critical growth factors, including IGFBP1 and PRL, is proposed to be the essential component of decidualization (Gellersen & Brosens 2014, Ng *et al.* 2020). Much research has focused on the effects of ASA therapy on early pregnancy and trophoblast cells (Bose *et al.* 2005, Panagodage *et al.* 2016, Ren *et al.* 2023) and later pregnancy (ACOG Committee Opinion No. 743, 2018). Pre-conception initiation of ASA has been shown to increase live births and reduce pregnancy loss among women with a history of pregnancy loss (Naimi *et al.* 2021). Nonetheless, there remains a paucity of research on the impact of ASA on ESCs and their function. This study is the first to examine the effect of ASA pre-treatment on ESC decidualization among participants with and without endometriosis.

## Materials and methods

### Chemicals and reagents

8-bromoadenosine 3′,5′-cyclic monophosphate sodium salt (cAMP), medroxyprogesterone acetate (MPA), and acetylsalicylic acid (ASA or aspirin) were purchased from Sigma-Aldrich (USA).

### Menstrual effluent (ME) samples and isolation of endometrial stromal cells

Women of reproductive age (22–40 years with a mean age of 32.3 years (SD: 5.5)) who were not pregnant, breastfeeding, or taking oral contraceptives and were menstruating and willing to provide ME samples were recruited and consented through the IRB- approved ROSE study at Northwell Health (13-376A, https://feinstein.northwell.edu/institutes-researchers/institute-molecular-medicine/robert-s-boas-center-for-genomics-and-human-genetics/rose-research-outsmarts-endometriosis). All participants in this study provided written informed consent before donating their ME (including ME-derived cells that were isolated and cultured) for ongoing and future studies related to endometriosis and uterine health. Women with histologically confirmed endometriosis (determined following laparoscopic surgery and documented in a pathology report) were recruited and enrolled as ‘endometriosis’ participants. Control participants who self-reported no history suggestive of a diagnosis of endometriosis were recruited and enrolled. ME was collected, de-identified, and processed to culture and cryopreserve ME-derived ESCs, as previously described (Warren *et al.* 2018, Delenko *et al.* 2024). Briefly, participants collected their ME at home for 4–8 h on the day of their heaviest menstrual flow (typically day 1 or 2 of the cycle) using a menstrual cup provided by DIVA International. After collection, they shipped their ME overnight at 4°C to the laboratory for processing. De-identified ME samples (500 μL per T-75 flask) were plated in growth media (DMEM high-glucose media containing 10% MSC-FBS, Normocin (a novel antibiotic formulation that protects against mycoplasma, bacterial, and fungal contaminations), and penicillin–streptomycin–glutamine (PSQ)) to culture ME-ESCs. ME-ESC cultures were monitored over time by visualization under a light microscope and phenotyping as in Warren *et al.* (2018). When mostly confluent, passage 0 and 1 ME-ESCs were cryopreserved. Formal sample size calculations were not performed as this is the first known study to examine the impact of ASA on ME-derived stromal cells, and there are no prior studies to inform effect size calculations.

### Culture of menstrual effluent (ME)-derived endometrial stromal cells

De-identified cryopreserved ME-derived ESCs (ME-ESCs) (passages 2–4) from 12 participants (8 control and 4 endometriosis) were defrosted, cultured, and expanded in growth media at 37°C/5%CO_2_, as previously described (Warren *et al.* 2018, Nayyar *et al.* 2020, Delenko *et al.* 2024).

### Decidualization assays

Decidualization assays were initiated using confluent ME-ESC cultures collected from controls (*n* = 8) and endometriosis participants (*n* = 4). For each participant, 1.5 × 10^4^ ME-ESCs in 200 μL of growth media (as described above) were plated per well in a 96-well plate and allowed to grow at 37°C/5%CO_2_ until confluence (∼1–2 days). Media was replaced with decidualization media (DMEM high-glucose media containing 2% MSC-FBS, Normocin, and PSQ). The next day, confluent ME-ESCs were pre-treated with vehicle or ASA (1–2.5 mM) for 4 h. ME-ESCs were then stimulated with vehicle (*n* = 3 wells per participant), 0.5 mM cAMP alone (*n* = 3 wells per participant), or 0.5 mM cAMP + 10^−7^ M MPA (*n* = 3 wells per participant), as previously described (Nayyar *et al.* 2020, Delenko *et al.* 2024). After 48 h, culture supernatants were collected after brief centrifugation and cell-free supernatants were analyzed for decidualization markers, IGFBP1 or PRL, using Human IGFBP1 and PRL DuoSet ELISA Kits (R&D Systems®, USA), respectively, according to the manufacturer’s directions and as previously reported (Warren *et al.* 2018, Nayyar *et al.* 2020, Delenko *et al.* 2024). All standards and samples were tested in triplicate (technical replicates) by ELISA following the manufacturer’s instructions. Immediately after adding the stop solution (Fisher Scientific), optical densities/absorbance readings were obtained at 450 nm/570 nm using an ELISA plate reader (MRX plate reader, Dynex/Dynatech), and values were extrapolated using IGFBP1 and PRL standard curves. After determining the average IGFBP1 and PRL protein values for each set of wells, data for cAMP-treated and cAMP + MPA-treated cells were normalized to fold-change using vehicle-treated decidualized cells as 1.

### Aspirin (ASA) dose selection

ASA doses were chosen based on published studies using stromal or fibroblast cells or differentiation assays (Sakurada *et al.* 1996, Ricchi *et al.* 1997, Li *et al.* 2017, Funke *et al.* 2024), as well as a dose–response curve obtained by pre-treating ESCs (obtained from three participants) for 4 h prior to decidualization (assessed by IGFBP1 production 48 h later) as described above (Supplementary Fig. 1, see the section on Supplementary materials given at the end of the article).

### Cytotoxicity assay

Neutral red uptake assays were used to assess potential cytotoxicity of ASA treatment of ME-ESCs obtained from participants with endometriosis (*n* = 4) and without endometriosis (*n* = 6), as previously described (Repetto *et al.* 2008).

### Proliferation assay

Proliferation assays were initiated under basal (non-decidualization) conditions using ME-ESCs from 6 control and 4 endometriosis participants. For each participant, 1.5 × 10^3^ ME-ESCs in 200 μL of growth media (as described above) were plated per well in a 96-well plate. ME-ESCs were incubated overnight at 37°C/5%CO_2_ and then treated with either vehicle or ASA (1–2.5 mM). After 72 h, the cells were analyzed using the CyQUANT^TM^ Cell Proliferation Assay Kit (Thermo Fisher, USA), according to the manufacturer’s instructions. All samples were tested in at least quadruplicate (technical replicates) for proliferation following the manufacturer’s instructions. Data were normalized to fold-change (cell number) using vehicle-treated cells as 1.

### Western blot analyses to identify potential target proteins

ESCs (p3-4) from healthy controls were plated at 2.5 × 10^5^ cells/mL (2 mL/well) in growth media in a 6-well plate (2 wells/condition). When confluent, growth media was replaced with decidualization media. The next day, ESCs were treated with either vehicle or ASA (2.5 mM). After 4 h, ESCs were washed with cold PBS and lysed with RIPA buffer containing protease and phosphatase inhibitors (Halt™ Protease and Phosphatase Inhibitor Cocktail, Thermo Fisher) and lysates were analyzed by western blotting, as in Delenko *et al.* (2024) and Delenko *et al.* (2025). Briefly, pre-stained molecular weight standards or ESC lysates (35–40 μg protein/lane) were separated by electrophoresis, transferred onto Immobilon FL-PVDF membranes, and immunoblotted sequentially with primary antibodies: phospho-AKT (Ser473, S473) monoclonal, AKT polyclonal, COX-1 monoclonal, COX-2 monoclonal, and GAPDH monoclonal antibodies (1:1,000) (Cell Signaling Technology, USA), as per manufacturer’s instructions. Band densities were quantified using NIH ImageJ and normalized to GAPDH protein levels and to specific total protein where applicable (e.g. phospho-AKT:total AKT) on the same blots.

### Statistical analysis

GraphPad Prism version 10.2.3 (403) (GraphPad Software Inc., https://www.graphpad.com/scientific-software/prism/) was used for all statistical analyses. Because data did not meet the assumptions of normality and equal variance, non-parametric tests were employed. For data sets with three or more groups, we used the Kruskal–Wallis test (a non-parametric ANOVA equivalent), and for all significant results, post hoc testing was performed using Dunn’s multiple comparison test. For groups of two, Mann–Whitney U tests were used. *P* < 0.05 was considered significant. For western blotting data, groups were compared using the Wilcoxon signed-rank test, a non-parametric test for paired samples.

## Results

Our results demonstrate that ASA enhances ESC decidualization markers and reduces ESC proliferation, without inducing significant cytotoxicity. There was no difference in the effects of ASA on ESC outcomes when comparing those cells obtained from women with vs without endometriosis. Note that limited numbers of endometriosis-ESCs were analyzed (*n* = 4).

### ASA improves decidualization marker production

**Figure 1:**
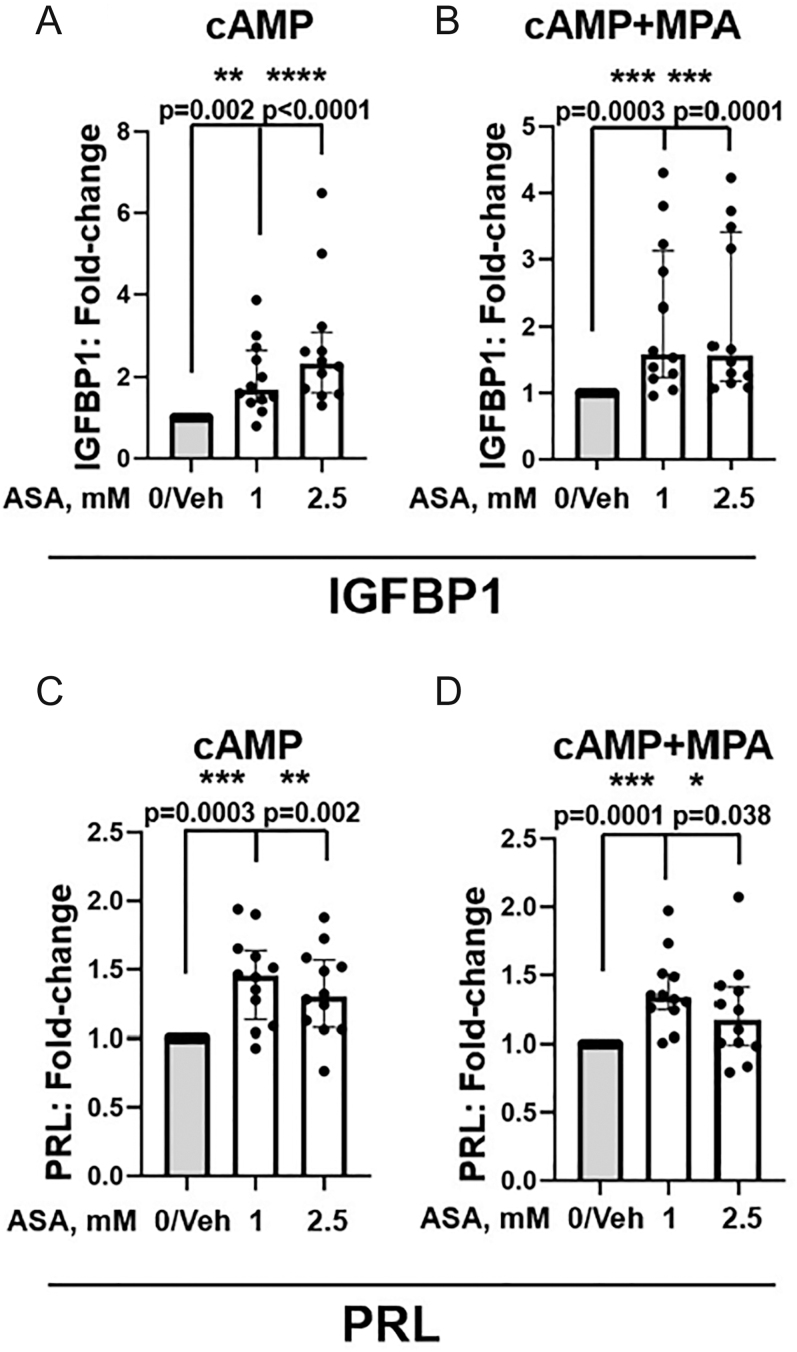
(A, B, C, D) Aspirin (ASA) enhances decidualization markers of endometrial stromal cells (ESCs). Treatment of ESCs with ASA (1–2.5 mM) prior to stimulation with either (A) cAMP alone or (B) cAMP + MPA enhances decidualization markers, as determined by IGFBP1 protein levels by ELISA when compared to vehicle (Veh)-treated ESCs. Treatment of ESCs with ASA (1–2.5 mM) prior to stimulation with either (C) cAMP alone or (D) cAMP + MPA enhances decidualization markers, as determined by PRL protein levels by ELISA compared to vehicle (Veh)-treated ESCs. Each dot represents data from ESCs isolated from one participant. Significance was determined by the Kruskal–Wallis test with post hoc Dunn’s multiple comparison test; *P*-values are shown. *P < 0.05, **P < 0.01, ***P < 0.001, ****P < 0.0001.

Decidualization assays revealed enhanced levels of ESC decidualization markers IGFBP1 and PRL after ASA treatment at the doses studied (Fig. 1A, B, C, D). In particular, ASA (1 mM) pre-treatment of ESCs increased production of decidualization marker IGFBP1 compared to vehicle in both cAMP-treated (median = 1.69 (IQR: 1.39, 2.65), *P* = 0.002) and cAMP + MPA-treated (median = 1.59 (IQR: 1.24, 3.13), *P* = 0.0003) ESCs (Fig. 1A and B). The addition of ASA (2.5 mM) increased production of decidualization marker IGFBP1 compared to vehicle treatment in both cAMP-treated (median = 2.33 (IQR: 1.62, 3.09), *P* < 0.0001) and cAMP + MPA-treated (median = 1.57 (IQR: 1.18, 3.41), *P* = 0.0001) ESCs. Likewise, ASA-enhanced decidualization marker PRL at both 1 and 2.5 mM ((median = 1.45 (IQR: 1.13, 1.64), *P* = 0.0003) and (median 1.30 (IQR 1.08, 1.57), *P* = 0.002), respectively) for cAMP induction (Fig. 1A and B). Similarly, PRL production following cAMP + MPA stimulation also increased, supporting enhanced decidualization following ASA 1 and 2.5 mM pre-treatment ((median = 1.34 (IQR: 1.25, 1.50), *P* = 0.0001) and (median = 1.17 (IQR: 0.99, 1.41), *P* = 0.038), respectively) (Fig. 1C and D). There were no statistically significant differences between the responsiveness of participants’ ESCs to ASA regardless of disease state (control vs endometriosis) (see Supplementary Fig. 2A, B, C, D, E, F, G, H). The direct comparisons of the raw data for IGFBP1 and PRL values for vehicle vs ASA 2.5 mM on a per-subject basis show similar results (see Supplementary Fig. 3A, B, C, D, E, F, G, H).

### Effects of ASA on ESC cytotoxicity

**Figure 2:**
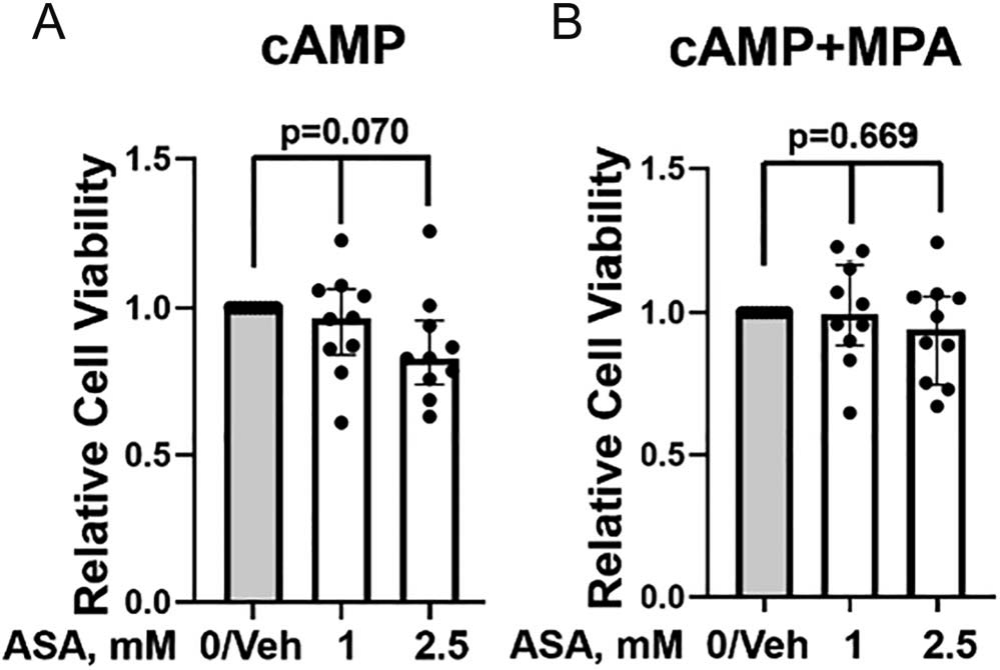
(A and B) Effects of aspirin (ASA) on ESC cytotoxicity. Treatment of ESCs with ASA (1–2.5 mM) prior to stimulation with either (A) cAMP alone or (B) cAMP + MPA was not cytotoxic, as determined by neutral red cellular assay when compared to vehicle (Veh)-treated ESCs. Each dot represents data from ESCs isolated from one participant. The Kruskal–Wallis test was used to determine significance; *P*-values are shown. Note: *post hoc* testing was not performed in the absence of significant findings.

Although limited cytotoxicity was observed following ASA treatment of ESCs (*n* = 10 per group), no significant cytotoxicity was found (Fig. 2A and B). As shown in Fig. 2A, Kruskal–Wallis testing for the three groups of ESCs treated with cAMP (vehicle, 1 mM ASA (median = 0.97 (IQR: 0.84, 1.06)), and 2.5 mM ASA (median = 0.83 (IQR: 0.74, 0.96))) did not reveal statistically significant results (*P* = 0.07). Therefore, post hoc testing to identify specific differences among the three groups in Fig. 2A was not performed. Similarly, as shown in Fig. 2B for ESCs treated with cAMP + MPA, Kruskal–Wallis testing for the three groups (vehicle, 1 mM ASA (median = 1.00 (IQR: 0.88, 1.17)), and 2.5 mM ASA (median = 0.94 (IQR: 0.75, 1.06))) did not reveal statistically significant results (*P* = 0.669). Thus, post hoc testing was not performed.

Aspirin-treated ESCs showed no significant cytotoxicity vs vehicle treatment regardless of whether they were obtained from control (*n* = 6) or endometriosis participants (*n* = 4, a limited sample size) (see Supplementary Fig. 4A, B, C, D). Direct comparisons of the raw data for cytotoxicity for vehicle vs ASA 2.5 mM on a per-participant basis showed no significant cytotoxic effects (see Supplementary Fig. 5A, B, C, D).

### High-dose ASA reduces ESC proliferation

**Figure 3:**
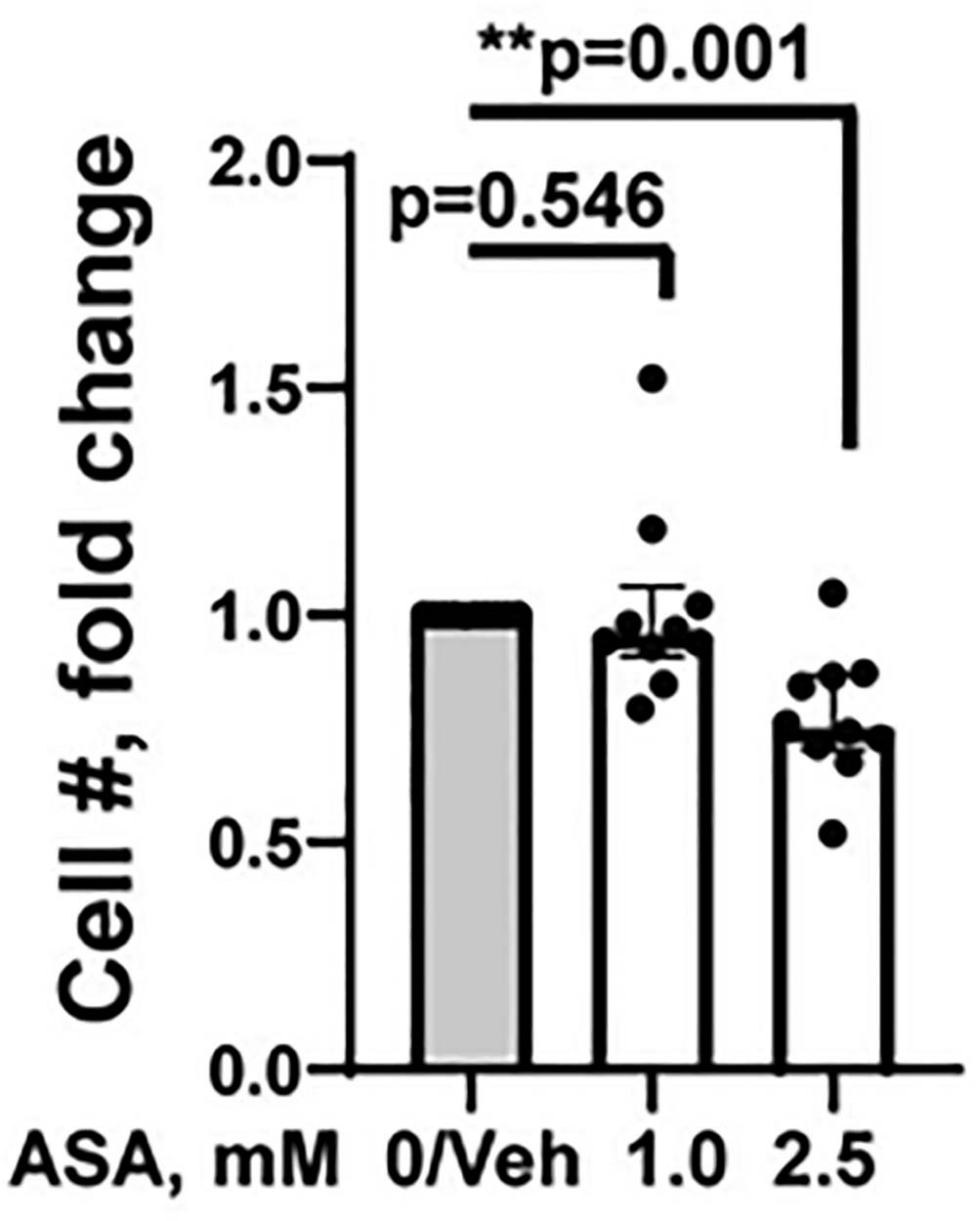
High-dose aspirin (ASA) reduces ESC proliferation. ASA treatment (2.5 mM) decreased proliferation of ESCs when compared to vehicle (Veh)-treated ESCs. There was no difference in proliferation of ESCs treated with ASA (1 mM) compared to vehicle (Veh)-treated ESCs. Each dot represents data from ESCs isolated from one participant. Significance was determined by the Kruskal–Wallis test with *post hoc* Dunn’s multiple comparison test; *P*-values are shown. **P < 0.01.

While ASA (1 mM) treatment of ESCs did not significantly affect their proliferation (median = 1.00 (IQR: 0.91, 1.06), *P* = 0.546), the higher dose of ASA (2.5 mM) significantly reduced proliferation compared to vehicle treatment (median = 0.75 (IQR: 0.70, 0.87), *P* = 0.001) (Fig. 3).

Although limited numbers of endometriosis-ESCs were tested (*n* = 4), there were no statistically significant differences in proliferation at either dose of ASA regardless of disease state (control vs endometriosis) (see Supplementary Fig. 6A and B). See Supplementary Fig. 7, which provides direct comparisons of the raw proliferation data for vehicle vs ASA 2.5 mM on a per-subject basis; 2.5 mM ASA treatment significantly reduced ESC proliferation.

### High-dose ASA reduces AKT phosphorylation

**Figure 4:**
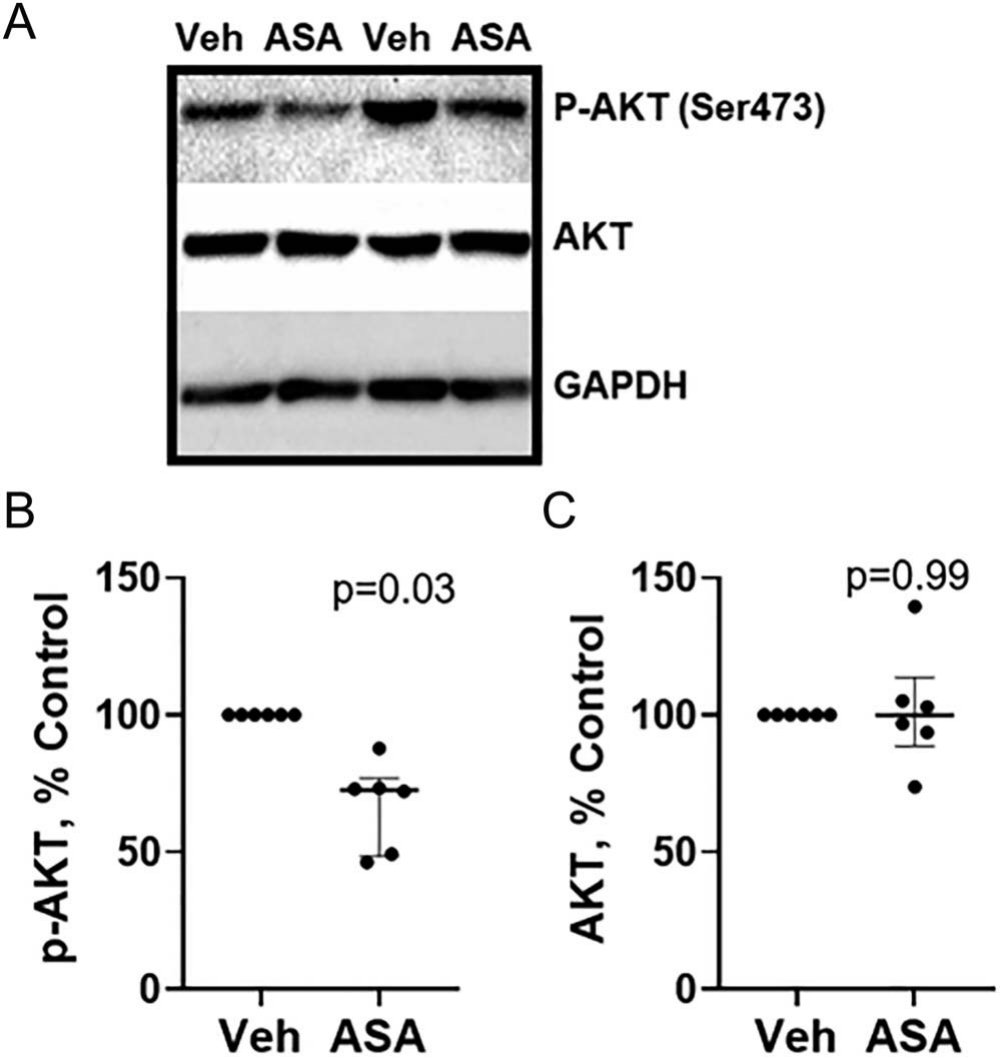
(A, B, C) High-dose aspirin (ASA) reduces AKT phosphorylation without affecting total AKT expression. ASA treatment (2.5 mM) of control ESCs decreased AKT phosphorylation (p-AKT) when compared to vehicle (Veh)-treated ESCs, without altering total AKT expression. (A) Representative blots showing phospho-AKT (P-AKT), total AKT, and GAPDH (loading control) from ESCs obtained from two subjects. (B and C) Quantification of band densities for (B) p-AKT and (C) total (AKT). Each dot represents data from ESCs isolated from one participant (*n* = 6 participants total). Significance was determined by the Wilcoxon signed-rank test for paired samples; *P*-values are shown.

Compared to vehicle treatment, ASA (2.5 mM) treatment significantly reduced AKT phosphorylation by ESCs by approximately 30% (*P* = 0.03), as determined by western blotting (Fig. 4). See Supplementary Fig. 8 for full blots and density calculations. By contrast, ASA (2.5 mM) treatment had no effect on AKT expression by ESCs (Fig. 4). Neither COX-1 nor COX-2 expression by ESCs was detected. See Supplementary Fig. 9 for COX-1 and COX-2 full western blots, showing the absence of bands of expected molecular weight.

## Discussion

This is the first study reporting the effect of ASA on human ESC decidualization. Our results support that ASA pre-treatment enhances ESC decidualization, as evidenced by the production of two decidualization markers (IGFBP1 and PRL), and reduces ESC proliferation, without inducing significant cytotoxicity. Although limited numbers of endometriosis-ESCs were analyzed, comparable results were observed in ESCs obtained from women with and without endometriosis. Mechanistic studies support that ASA reduces AKT phosphorylation or AKT signaling, consistent with the inhibition of AKT activation observed during decidualization (Lee *et al.* 2016, Fabi *et al.* 2017).

Although historically, ASA has been utilized as an adjunct in early pregnancy, data are mixed regarding its effectiveness (Siristatidis *et al.* 2016, Wang *et al.* 2017, Glujovsky *et al.* 2020). ASA use is recommended during early pregnancy for many patients to reduce the risk of preeclampsia (ACOG Committee Opinion No. 743. 2018). One study supports the initiation of ASA prior to conception to improve pregnancy outcomes (Naimi *et al.* 2021). This randomized trial demonstrated that preconception ASA treatment was associated with an increased live birth rate and reduced pregnancy loss among a cohort of women with a history of pregnancy loss (Naimi *et al.* 2021). However, the mechanism(s) by which ASA treatment may improve pregnancy outcomes remains unknown. Likewise, a systematic review and meta-analysis of randomized controlled trials demonstrated that ASA use in IVF (*in vitro* fertilization)/ICSI (intracytoplasmic sperm injection) cycles was associated with improved clinical pregnancy rates (Wang *et al.* 2017). However, other studies have not yielded similar findings (Davar *et al.* 2020, He *et al.* 2023). Despite these reports, mechanistic studies examining how ASA affects human ESCs, *in vitro* and *in vivo*, are lacking.

In humans, decidualization is an essential, well-regulated differentiation process that precedes successful implantation (Okada *et al.* 2018), and this process is accompanied by reduced ESC proliferation along with reduced AKT phosphorylation (also known as AKT signaling) (Lee *et al.* 2016, Fabi *et al.* 2017). Decidualized ESCs contribute to the microenvironment at the endometrial–trophoblast interface and play multiple essential roles in the complex interaction between the endometrium and the attaching embryo (Okada *et al.* 2018). Although improper decidualization has been reported in the settings of infertility and recurrent pregnancy loss (Teklenburg *et al.* 2010, Okada *et al.* 2018, Dambaeva *et al.* 2020), endometriosis (Nayyar *et al.* 2020), and preeclampsia (Garrido-Gomez *et al.* 2017) and reduced decidualized cells at the implantation site are known to predispose to early pregnancy failure (Muter *et al.* 2021), this process is still incompletely understood, and much remains unknown.

The majority of prior studies of human ESCs have relied on invasive endometrial biopsies for collecting ESCs, and this procedure has been mainly restricted to those patients undergoing biopsies for uterine-related conditions or via hysterectomies, many of which occur outside the reproductive years. Thus, little is known about the functional responses of ESCs in healthy women. Our ‘population-based’ approach using ME-derived ESCs is non-invasive and significantly expands the collection of ESCs to include most menstruating participants with or without uterine health conditions and provides larger samples (and hence, larger yields of ESCs for experiments) than typical endometrial biopsies.

Our results indicate significant increases in decidualization markers, IGFBP1 and PRL, following treatment with ASA (1–2.5 mM). IGFBP1 and PRL are two of the most well-established and commonly used biomarkers of human ESC decidualization *in vitro* (Gellersen & Brosens 2014, Okada *et al.* 2018, Rodriguez-Caro *et al.* 2019); their relative concentrations measured in the culture supernatants of decidualizing ESCs represent the functional cell-based secretion of nutritive growth factors by decidualizing ESCs required for human embryo implantation and successful pregnancy (Gellersen & Brosens 2014). Assessment of both IGFBP1 and PRL added rigor and certainty to our results. Given previously published findings that, in decidualizing ESCs, IGFBP1 production increases more rapidly than PRL production (Fluhr *et al.* 2006), it was anticipated that IGFBP1 results may be more robust compared to PRL results given the timing of decidualization marker assessment (48 h after cAMP ± MPA). In addition, our proliferation assay results revealed a statistically significant reduction in ESC proliferation when treated with the higher dose of ASA (2.5 mM, *P* = 0.001). Evidence supports the reciprocal relationship between cell proliferation and cell differentiation and that cell cycle arrest accompanies cellular differentiation (Wille & Scott 1986, Rogers & Abberton 2003, Kolly *et al.* 2005, Logan *et al.* 2012). Accordingly, many factors or agents that reduce ESC proliferation (Das 2009, Mestre Citrinovitz *et al.* 2020, Lyu *et al.* 2023, Delenko *et al.* 2024) promote ESC decidualization (e.g., quercetin). By contrast, inhibitors of decidualization may promote ESC proliferation (Le *et al.* 2017). High-dose ASA (2.5 mM) significantly inhibited proliferation and showed limited effects on cell viability following cAMP stimulation (Fig. 2A). However, the effect on viability was not significant. Moreover, high-dose ASA (2.5 mM) had no significant cytotoxic effect on ESCs treated with cAMP + MPA (Fig. 2B), which better reflects the *in vivo* decidualization response (Doi-Tanaka *et al.* 2024). There is emerging evidence that endometrial compaction, which can be detected by ultrasound during the secretory phase when decidualization occurs, may predict successful implantation, although data on live birth outcomes remain lacking (Al-Lamee *et al.* 2024). Our study demonstrated significant increases in decidualization markers following pre-treatment of ESCs with ASA (1–2.5 mM), which could become clinically relevant if replicated *in vivo*. This assessment of the *in vivo* effects of ASA on ME-derived ESCs is possible in a clinical study where ESCs are collected and analyzed for decidualization response *ex vivo* pre- and post-ASA administration *in vivo*, along with assessment of ASA or its metabolites in ME (representative of the local uterine environment).

Regarding the mechanisms underlying the deciduogenic activity of ASA, ASA irreversibly inhibits both COX-1 and COX-2 enzyme activity, with stronger affinity for COX-1 inhibition (Mitchell *et al.* 1993, Qureshi & Dua 2024). PGI_2_ and PGE_2_ production are dependent upon arachidonic acid cleavage by COX-2 (Gnecco *et al.* 2019), and prostaglandin secretion by ESCs promotes enhanced responsiveness to progesterone via cAMP activation, resulting in increased decidualization (Gnecco *et al.* 2019). A prior study noted decreased decidualization *in vivo* when using celecoxib to block COX-2 expression in rats (Sookvanichsilp & Pulbutr 2002). Although other studies have demonstrated COX-1 and COX-2 activity in human ESCs (Shaw *et al.* 1994, Wang *et al.* 2006), we were unable to detect protein expression by western blotting analysis, which precluded further assessing preferential inhibition of COX-1 (or COX-2) protein expression as a mechanism of action to explain our results.

ASA has been shown to lead to loss of AKT phosphorylation in both rodent (Liu *et al.* 2017) and human cell lines (Cho *et al.* 2013, Chen *et al.* 2020, Peng *et al.* 2023). Interestingly, ASA-induced loss of AKT phosphorylation occurs in a COX-1-dependent manner in human ovarian cancer cells, which are known to express higher levels of COX-1 rather than COX-2 (Cho *et al.* 2013).

Because our study is the first to examine the impact of ASA on ESC decidualization markers, we hypothesize that the effect of ASA on decidualization may be due to the inhibition of COX-1 and subsequent reduced AKT phosphorylation as described by numerous prior studies (Cho *et al.* 2013, Liu *et al.* 2017, Chen *et al.* 2020, Peng *et al.* 2023). However, we were unable to detect COX-1 or COX-2 protein expression by ESCs under our culture conditions.

Although the mechanism of action for ASA’s impact on ESCs was not the primary focus of our study, our western blotting analysis of signaling proteins implicated in decidualization demonstrated loss of AKT phosphorylation after exposure to ASA. COX-1 inhibitors can also lead to loss of AKT phosphorylation (Altintop *et al.* 2023), and loss of AKT phosphorylation reduces the expression of collagenI (Bujor *et al.* 2008). ASA has been shown to reduce expression of collagen 1A1, α-smooth muscle actin (αSMA), and fibronectin and inhibit endometrial fibrosis (Zhang Z *et al.* 2020) and improve endometrial thickness in some studies (Lebovitz & Orvieto 2014, Li *et al.* 2023); however, other studies report no such effects (Davar *et al.* 2020). Interestingly, *COL1A1* mRNA expression is increased in ME-derived ESCs derived from patients with endometriosis when compared to ESCs collected from unaffected controls (Shih *et al.* 2022).

Prior studies demonstrated that downregulation of phospho-Ser473-AKT (loss of AKT signaling) promotes decidualization (Fabi *et al.* 2017), while persistent expression of phospho-Ser473-AKT inhibits decidualization (Yin *et al.* 2012). Based on these and other studies showing that loss of AKT phosphorylation enhances decidualization (Delenko *et al.* 2024, 2025), our data support that ASA promotes loss of AKT phosphorylation, and this is accompanied by enhanced decidualization biomarker expression. Our findings are consistent with recent studies describing the pro-deciduogenic activity of quercetin mediated through the AKT signaling pathway (Park *et al.* 2019, Delenko *et al.* 2024, 2025).

This study has several notable strengths, including utilizing primary human ESCs and measuring both IGFBP1 and PRL for assessing decidualization, analyzing the outcome measures in primary ESCs obtained from both unaffected controls and women with endometriosis, and being the first of its kind. In addition, our western blot analysis suggests that ASA (2.5 mM) inhibits AKT phosphorylation to promote ESC decidualization. Limitations of our study include the use of an *in vitro* model system, relatively small sample sizes (which may include some heterogeneity in responses among participants in our study), limited knowledge of participants’ reproductive/fertility and medical histories, and not fully elucidating the underlying mechanism(s) of action. Finally, although considered hallmark features of decidualization, relying on two of the most well-established markers (IGFBP1 and PRL) at the 48 h time point may not fully capture the complete process of decidualization. Despite these limitations, this study is the first to report the enhancing effects of ASA on ESC decidualization markers and the inhibitory effects of ASA on ESC proliferation. Future research should include a larger sample size, include healthy controls and endometriosis patients, including infertility patients with defined fertility and reproductive histories, and aim to further elucidate how ASA regulates AKT phosphorylation in ESCs to better define the mechanism(s) of action of ASA.

In conclusion, our results support that ASA, at 1 and 2.5 mM, significantly enhanced decidualization biomarker expression by human ESCs without inducing significant cytotoxicity. In addition, high-dose ASA (2.5 mM) significantly reduced ESC proliferation and reduced AKT phosphorylation. These findings are consistent with prior studies showing that stromal cell cycle arrest is associated with decidualization (Logan *et al.* 2012), and reduced AKT signaling positively regulates decidualization (Fabi *et al.* 2017), while overactivation of AKT signaling disrupts decidualization (Yin *et al.* 2012). We observed no difference in ESC responsiveness based on control vs endometriosis status of the participants. Because endometriosis has been associated with impaired decidualization (Klemmt *et al.* 2006, Minici *et al.* 2008, Warren *et al.* 2018, Nayyar *et al.* 2020), further *in vitro* studies assessing ASA’s impact on ESCs are warranted among this patient population. In addition, while our western blot analysis suggests that ASA may act through the AKT pathway to affect decidualization, further research into the precise mechanism of action using pharmacological inhibition or genetic manipulation is warranted. Before utilizing ASA as an adjunct to improve fertility and reproductive outcomes in patients with endometriosis and infertility, future studies are required to explore the effects of ASA *in vivo* at doses typically utilized in clinical practice. Our results should prompt further mechanistic investigations into ASA’s effect on ESC function and ASA’s potential to enhance decidualization and improve fertility and pregnancy outcomes.

## Supplementary materials







## Declaration of interest

ERM is an active-duty member of the US Army. RHG is a peer reviewer for UpToDate and a Medical Expert at Roon. XX, PC, NH, RAB, PKG and CNM have nothing to disclose. 

## Funding

DIVA International provided the menstrual cups for the ROSE study.

## Author contribution statement

ERM conceived the study, curated the data, performed formal analysis and investigation, and wrote, reviewed, and edited the manuscript. XX, PC, and H curated the data, performed investigation, and wrote, reviewed, and edited the manuscript. RAB, RHG, PKG supervised the study and wrote, reviewed, and edited the manuscript. CNM conceived the study, designed the methodology, curated the data, performed formal analysis and investigation, supervised the study, and wrote, reviewed, and edited the manuscript.

## Data availability

The data underlying this article will be shared on reasonable request to the corresponding author.

## Disclaimer

The views and information presented are those of the authors and do not represent the official position of the US Army Medical Center of Excellence, the US Army Training and Doctrine Command, or the Department of the Army, Department of Defense, or US Government.

## References

[bib1] 2018 ACOG committee opinion no. 743: low-dose aspirin use during pregnancy. Obstet Gynecol 132 e44–e52. (10.1097/AOG.0000000000002708) 29939940

[bib2] Al-Lamee H, Stone K, Powell SG, et al. 2024 Endometrial compaction to predict pregnancy outcomes in patients undergoing assisted reproductive technologies: a systematic review and meta-analysis. Hum Reprod Open 2024 hoae040. (10.1093/hropen/hoae040) 38993630 PMC11239225

[bib3] Altıntop MD, Akalın Çiftçi G, Yılmaz Savaş N, et al. 2023 Discovery of small molecule COX-1 and Akt inhibitors as Anti-NSCLC agents endowed with anti-inflammatory action. Int J Mol Sci 24 2648. (10.3390/ijms24032648) 36768971 PMC9916685

[bib4] Barragan F, Irwin JC, Balayan S, et al. 2016 Human endometrial fibroblasts derived from mesenchymal progenitors inherit progesterone resistance and acquire an inflammatory phenotype in the endometrial niche in endometriosis. Biol Reprod 94 118. (10.1095/biolreprod.115.136010) 27075616 PMC4939744

[bib5] Boelig RC, Kaushal G, Rochani A, et al. 2024 Aspirin pharmacokinetics and pharmacodynamics through gestation. Am J Obstet Gynecol 231 344.e1–344.e16. (10.1016/j.ajog.2023.12.028) PMC11193839 38145726

[bib6] Bose P, Black S, Kadyrov M, et al. 2005 Heparin and aspirin attenuate placental apoptosis in vitro: implications for early pregnancy failure. Am J Obstet Gynecol 192 23–30. (10.1016/j.ajog.2004.09.029) 15671997

[bib7] Bujor AM, Pannu J, Bu S, et al. 2008 Akt blockade downregulates collagen and upregulates MMP1 in human dermal fibroblasts. J Invest Dermatol 128 1906–1914. (10.1038/jid.2008.39) 18323784

[bib8] Cai X, Xu M, Zhang H, et al. 2022 Endometrial stromal PRMT5 plays a crucial role in decidualization by regulating NF-κB signaling in endometriosis. Cell Death Discov 8 408. (10.1038/s41420-022-01196-x) 36195592 PMC9532444

[bib9] Chen Z, Wang C, Dong H, et al. 2020 Aspirin has a better effect on PIK3CA mutant colorectal cancer cells by PI3K/Akt/Raptor pathway. Mol Med 26 14. (10.1186/s10020-020-0139-5) 32000660 PMC6993447

[bib10] Cho M, Kabir SM, Dong Y, et al. 2013 Aspirin blocks EGF-stimulated cell viability in a COX-1 dependent manner in ovarian cancer cells. J Cancer 4 671–678. (10.7150/jca.7118) 24155779 PMC3805995

[bib11] Cinar O, Seval Y, Uz YH, et al. 2009 Differential regulation of Akt phosphorylation in endometriosis. Reprod Biomed Online 19 864–871. (10.1016/j.rbmo.2009.10.001) 20031030

[bib12] Connell MT, Sjaarda LA, Radin RG, et al. 2017 The effects of aspirin in gestation and reproduction (EAGeR) trial: a story of discovery. Semin Reprod Med 35 344–352. (10.1055/s-0037-1606384) 29036741 PMC6234510

[bib13] Dambaeva S, Bilal M, Schneiderman S, et al. 2020 Decidualization score identifies an endometrial dysregulation in samples from women with recurrent pregnancy losses and unexplained infertility. Fertil Sterility Rep 2 95–103. (10.1016/j.xfre.2020.12.004) PMC8244268 34223279

[bib14] Das SK 2009 Cell cycle regulatory control for uterine stromal cell decidualization in implantation. Reproduction 137 889–899. (10.1530/REP-08-0539) 19307426

[bib15] Davar R, Pourmasumi S, Mohammadi B, et al. 2020 The effect of low-dose aspirin on the pregnancy rate in frozen-thawed embryo transfer cycles: a randomized clinical trial. Int J Reprod Biomed 18 693–700. (10.18502/ijrm.v13i9.7664) 33062915 PMC7521165

[bib16] Delenko J, Xue X, Chatterjee PK, et al. 2024 Quercetin enhances decidualization through AKT-ERK-p53 signaling and supports a role for senescence in endometriosis. Reprod Biol Endocrinol 22 100. (10.1186/s12958-024-01265-z) 39118090 PMC11308242

[bib17] Delenko J, Hyman N, Chatterjee PK, et al. 2025 Targeting cellular senescence to enhance human endometrial stromal cell decidualization and inhibit their migration. Biomolecules 15 873. (10.3390/biom15060873) 40563513 PMC12191167

[bib18] Doi-Tanaka Y, Tamura I, Shiroshita A, et al. 2024 Differential gene expression in decidualized human endometrial stromal cells induced by different stimuli. Sci Rep 14 7726. (10.1038/s41598-024-58065-z) 38565619 PMC10987566

[bib19] ESHRE Working Group on Recurrent Implantation Failure, Cimadomo D, de Los Santos MJ, Griesinger G, et al. 2023 ESHRE good practice recommendations on recurrent implantation failure. Hum Reprod Open 2023 hoad023. (10.1093/hropen/hoad023) 37332387 PMC10270320

[bib20] Fabi F, Grenier K, Parent S, et al. 2017 Regulation of the PI3K/Akt pathway during decidualization of endometrial stromal cells. PLoS One 12 e0177387. (10.1371/journal.pone.0177387) 28475617 PMC5419658

[bib21] Fishman P, Falah‐Vaknine E, Sredni B, et al. 1995 Aspirin modulates interleukin‐3 production: an additional explanation for the preventive effects of aspirin in antiphospholipid antibody syndrome. J Rheumatol 22 1086–1090. (https://pubmed.ncbi.nlm.nih.gov/7674234/) 7674234

[bib22] Fluhr H, Krenzer S, Deperschmidt M, et al. 2006 Human chorionic gonadotropin inhibits insulin-like growth factor-binding protein-1 and prolactin in decidualized human endometrial stromal cells. Fertil Steril 86 236–238. (10.1016/j.fertnstert.2005.12.031) 16818038

[bib23] Funke S, Wiggenhauser PS, Grundmeier A, et al. 2024 Aspirin stimulates the osteogenic differentiation of human adipose tissue-derived stem cells in vitro. Int J Mol Sci 25 7690. (10.3390/ijms25147690) 39062933 PMC11277042

[bib24] Glujovsky D, Pesce R, Sueldo C, et al. 2020 Endometrial preparation for women undergoing embryo transfer with frozen embryos or embryos derived from donor oocytes. Cochrane Database Syst Rev 10 CD006359. (10.1002/14651858.CD006359.pub3) 20091592

[bib25] Garrido-Gomez T, Dominguez F, Quiñonero A, et al. 2017 Defective decidualization during and after severe preeclampsia reveals a possible maternal contribution to the etiology. Proc Natl Acad Sci U S A 114 E8468–E8477. (10.1073/pnas.1706546114) 28923940 PMC5635883

[bib26] Gellersen B & Brosens JJ 2014 Cyclic decidualization of the human endometrium in reproductive health and failure. Endocr Rev 35 851–905. (10.1210/er.2014-1045) 25141152

[bib27] Gnecco JS, Ding T, Smith C, et al. 2019 Hemodynamic forces enhance decidualization via endothelial-derived prostaglandin E2 and prostacyclin in a microfluidic model of the human endometrium. Hum Reprod 34 702–714. (10.1093/humrep/dez003) 30789661 PMC6443116

[bib28] He H, Qi D, Fang M, et al. 2023 The effect of short-term aspirin administration during programmed frozen-thawed embryo transfer on pregnancy outcomes and complications. J Clin Med 12 1064. (10.3390/jcm12031064) 36769712 PMC9918171

[bib29] Klemmt PA, Carver JG, Kennedy SH, et al. 2006 Stromal cells from endometriotic lesions and endometrium from women with endometriosis have reduced decidualization capacity. Fertil Steril 85 564–572. (10.1016/j.fertnstert.2005.08.046) 16500320 PMC1626574

[bib30] Kolly C, Suter MM & Müller EJ 2005 Proliferation, cell cycle exit, and onset of terminal differentiation in cultured keratinocytes: pre-programmed pathways in control of C-Myc and Notch1 prevail over extracellular calcium signals. J Invest Dermatol 124 1014–1025. (10.1111/j.0022-202X.2005.23655.x). Erratum in: *Journal of Investigative Dermatology* 2006 126 2734. 15854044

[bib31] Le A, Wang ZH, Dai XY, et al. 2017 Icaritin inhibits decidualization of endometrial stromal cells. Exp Ther Med 14 5949–5955. (10.3892/etm.2017.5278) 29285144 PMC5740763

[bib32] Lebovitz O & Orvieto R 2014 Treating patients with “thin” endometrium – an ongoing challenge. Gynecol Endocrinol 30 409–414. (10.3109/09513590.2014.906571) 24693854

[bib33] Lee II & Kim JJ 2014 Influence of AKT on progesterone action in endometrial diseases. Biol Reprod 91 63. (10.1095/biolreprod.114.119255) 25100707 PMC4435059

[bib34] Lee SY, Lee YY, Choi JS, et al. 2016 Phosphatidic acid induces decidualization by stimulating Akt-PP2A binding in human endometrial stromal cells. FEBS J 283 4163–4175. (10.1111/febs.13914) 27696687

[bib35] Li Y, Luo Z, Xu X, et al. 2017 Aspirin enhances the osteogenic and anti-inflammatory effects of human mesenchymal stem cells on osteogenic BFP-1 peptide-decorated substrates. J Mater Chem B 5 7153–7163. (10.1039/c7tb01732d) 32263906

[bib36] Li LN, Li XD & Du J 2023 The effect of aspirin on uterine arterial blood flow and endometrium in moderate and severe intrauterine adhesion after transcervical resection of adhesion: a systematic review and meta-analysis. J Matern Fetal Neonatal Med 36 2209818. (10.1080/14767058.2023.2209818) 37286223

[bib37] Liao Z, Tang S, Jiang P, et al. 2024 Impaired bone morphogenetic protein (BMP) signaling pathways disrupt decidualization in endometriosis. Commun Biol 7 227. (10.1038/s42003-024-05898-z) 38402336 PMC10894266

[bib38] Liu PP, Liu HH, Sun SH, et al. 2017 Aspirin alleviates cardiac fibrosis in mice by inhibiting autophagy. Acta Pharmacol Sin 38 488–497. (10.1038/aps.2016.143) 28216620 PMC5386311

[bib39] Logan PC, Steiner M, Ponnampalam AP, et al. 2012 Cell cycle regulation of human endometrial stromal cells during decidualization. Reprod Sci 19 883–894. (10.1177/1933719112438447) 22534328

[bib40] Lyu M, Gao W, Zhang L, et al. 2023 Hsa_circ_0001550 impairs decidualization by regulating the proliferation and apoptosis of endometrial stromal cells. Reprod Biomed Online 46 225–233. (10.1016/j.rbmo.2022.10.003) 36396534

[bib41] Mitchell JA, Akarasereenont P, Thiemermann C, et al. 1993 Selectivity of nonsteroidal antiinflammatory drugs as inhibitors of constitutive and inducible cyclooxygenase. Proc Natl Acad Sci U S A 90 11693–11697. (10.1073/pnas.90.24.11693) 8265610 PMC48050

[bib42] Mestre Citrinovitz AC, Langer L, Strowitzki T, et al. 2020 Resveratrol enhances decidualization of human endometrial stromal cells. Reproduction 159 453–463. (10.1530/REP-19-0425) 31990677

[bib43] Minici F, Tiberi F, Tropea A, et al. 2008 Endometriosis and human infertility: a new investigation into the role of eutopic endometrium. Hum Reprod 23 530–537. (10.1093/humrep/dem399) 18096563

[bib44] Muter J, Kong CS & Brosens JJ 2021 The role of decidual subpopulations in implantation, menstruation and miscarriage. Front Reprod Health 3 804921. (10.3389/frph.2021.804921) 36303960 PMC9580781

[bib45] Naimi AI, Perkins NJ, Sjaarda LA, et al. 2021 The effect of preconception-initiated low-dose aspirin on human chorionic gonadotropin-detected pregnancy, pregnancy loss, and live birth: per protocol analysis of a randomized trial. Ann Intern Med 174 595–601. (10.7326/M20-0469) 33493011 PMC9109822

[bib46] Nayyar A, Saleem MI, Yilmaz M, et al. 2020 Menstrual effluent provides a novel diagnostic window on the pathogenesis of endometriosis. Front Reprod Health 2 3. (10.3389/frph.2020.00003) 36304708 PMC9580670

[bib47] Ng SW, Norwitz GA, Pavlicev M, et al. 2020 Endometrial decidualization: the primary driver of pregnancy health. Int J Mol Sci 21 4092. (10.3390/ijms21114092) 32521725 PMC7312091

[bib48] Okada H, Tsuzuki T & Murata H 2018 Decidualization of the human endometrium. Reprod Med Biol 17 220–227. (10.1002/rmb2.12088) 30013421 PMC6046526

[bib49] Park S, Lim W, Bazer FW, et al. 2019 Quercetin inhibits proliferation of endometriosis regulating cyclin D1 and its target microRNAs in vitro and in vivo. J Nutr Biochem 63 87–100. (10.1016/j.jnutbio.2018.09.024) 30359864

[bib50] Panagodage S, Yong HE, Da Silva Costa F, et al. 2016 Low-dose acetylsalicylic acid treatment modulates the production of cytokines and improves trophoblast function in an in vitro model of early-onset preeclampsia. Am J Pathol 186 3217–3224. (10.1016/j.ajpath.2016.08.010) 27750048

[bib51] Peng J, Xiao X, Li S, et al. 2023 Aspirin alleviates pulmonary fibrosis through PI3K/AKT/mTOR-mediated autophagy pathway. Exp Gerontol 172 112085. (10.1016/j.exger.2023.112085) 36623738

[bib52] Qureshi O & Dua A 2024 COX inhibitors. (https://www.ncbi.nlm.nih.gov/books/NBK549795/). Retrieved on 21 November 2024.

[bib53] Ren Y, Zhao Y, Yang X, et al. 2023 Application of low dose aspirin in pre-eclampsia. Front Med 10 1111371. (10.3389/fmed.2023.1111371) PMC10030847 36968826

[bib54] Repetto G, del Peso A & Zurita JL 2008 Neutral red uptake assay for the estimation of cell viability/cytotoxicity. Nat Protoc 3 1125–1131. (10.1038/nprot.2008.75) 18600217

[bib55] Ricchi P, Pignata S, Di Popolo A, et al. 1997 Effect of aspirin on cell proliferation and differentiation of colon adenocarcinoma Caco-2 cells. Int J Cancer 73 880–884. (10.1002/(sici)1097-0215(19971210)73:6<880::aid-ijc20>3.0.co;2-7) 9399670

[bib56] Rodriguez-Caro H, Dragovic R, Shen M, et al. 2019 In vitro decidualization of human endometrial stromal cells in enhanced by seminal fluid extracellular vesicles. J Extracell Vesicles 8 1565262. (10.1080/20013078.2019.1565262) 30728921 PMC6352950

[bib57] Rogers PA & Abberton KM 2003 Endometrial arteriogenesis: vascular smooth muscle cell proliferation and differentiation during the menstrual cycle and changes associated with endometrial bleeding disorders. Microsc Res Tech 60 412–419. (10.1002/jemt.10279) 12567398

[bib58] Rubinstein M, Marazzi A & Polak de Fried E 1999 Low-dose aspirin treatment improves ovarian responsiveness, uterine and ovarian blood flow velocity, implantation, and pregnancy rates in patients undergoing in vitro fertilization: a prospective, randomized, double-blind placebo-controlled assay. Fertil Steril 71 825–829. (10.1016/s0015-0282(99)00088-6) 10231040

[bib59] Sakurada S, Kato T & Okamoto T 1996 Induction of cytokines and ICAM-1 by proinflammatory cytokines in primary rheumatoid synovial fibroblasts and inhibition by N-acetyl-L-cysteine and aspirin. Int Immunol 8 1483–1493. (10.1093/intimm/8.10.1483) 8921427

[bib60] Shaw KJ, Ng C & Kovacs BW 1994 Cyclooxygenase gene expression in human endometrium and decidua. Prostaglandins Leukot Essent Fatty Acids 50 239–243. (10.1016/0952-3278(94)90160-0) 8066098

[bib61] Shih AJ, Adelson RP, Vashistha H, et al. 2022 Single-cell analysis of menstrual endometrial tissues defines phenotypes associated with endometriosis. BMC Med 20 315. (10.1186/s12916-022-02500-3) 36104692 PMC9476391

[bib62] Siristatidis CS, Basios G, Pergialiotis V, et al. 2016 Aspirin for in vitro fertilisation. Cochrane Database Syst Rev 11 CD004832. (10.1002/14651858.CD004832.pub4) 27807847 PMC6463901

[bib63] Sookvanichsilp N & Pulbutr P 2002 Anti-implantation effects of indomethacin and celecoxib in rats. Contraception 65 373–378. (10.1016/s0010-7824(01)00322-5) 12057792

[bib64] St-Louis I, Singh M, Brasseur K, et al. 2010 Expression of COX-1 and COX-2 in the endometrium of cyclic, pregnant and in a model of pseudopregnant rats and their regulation by sex steroids. Reprod Biol Endocrinol 8 103. (10.1186/1477-7827-8-103) 20735829 PMC2936314

[bib65] Ohlsson Teague EMC, Print CG & Hull ML 2010 The role of microRNAs in endometriosis and associated reproductive conditions. Hum Reprod Update 16 142–165. (10.1093/humupd/dmp034) 19773286

[bib66] Teklenburg G, Salker M, Molokhia M, et al. 2010 Natural selection of human embryos: decidualizing endometrial stromal cells serve as sensors of embryo quality upon implantation. PLoS One 5 e10258. (10.1371/journal.pone.0010258) 20422011 PMC2858159

[bib67] Walsh SW & Strauss JF 3rd 2021 The road to low-dose aspirin therapy for the prevention of preeclampsia began with the placenta. Int J Mol Sci 22 6985. (10.3390/ijms22136985) 34209594 PMC8268135

[bib68] Wang H, Wen Y, Lake Polan M, et al. 2006 Regulation of cyclooxygenase activity in cultured endometrial stromal cells by granulocyte-macrophage colony-stimulating factor. Fertil Steril 85 1118–1124. (10.1016/j.fertnstert.2005.09.040) 16616083

[bib69] Wang L, Huang X, Li X, et al. 2017 Efficacy evaluation of low-dose aspirin in IVF/ICSI patients evidence from 13 RCTs: a systematic review and meta-analysis. Medicine 96 e7720. (10.1097/MD.0000000000007720) 28906358 PMC5604627

[bib70] Warren LA, Shih A, Renteira SM, et al. 2018 Analysis of menstrual effluent: diagnostic potential for endometriosis. Mol Med 24 1. (10.1186/s10020-018-0009-6) 30134794 PMC6016873

[bib71] Wille JJJ & Scott RE 1986 Suppression of tumorigenicity by the cell-cycle-dependent control of cellular differentiation and proliferation. Int J Cancer 37 875–881. (10.1002/ijc.2910370613) 3710616

[bib72] Yin X, Pavone ME, Lu Z, et al. 2012 Increased activation of the PI3K/AKT pathway compromises decidualization of stromal cells from endometriosis. J Clin Endocrinol Metab 97 E35–E43. (10.1210/jc.2011-1527) 22072736 PMC3251935

[bib73] Zhang Z, Li S, Deng J, et al. 2020 Aspirin inhibits endometrial fibrosis by suppressing the TGF-β1-Smad2/Smad3 pathway in intrauterine adhesions. Int J Mol Med 45 1351–1360. (10.3892/ijmm.2020.4506) 32323728 PMC7138280

