# Supplementary material for: Aspirin enhances endometrial decidualization markers in vitro among women with and without endometriosis
Source: Reprod Fertil. 2026 Mar 26;7(1):RAF250034. doi: 10.1530/RAF-25-0034 (PMC13034528; doi:10.1530/RAF-25-0034)
Supplement: Supplementary file 1 [file supplementary_figure_1-7.pdf]

Supplementary Figure 1 Dose Response Curve

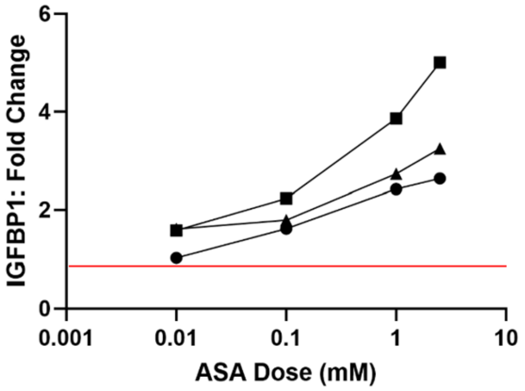

Supplementary Figure 1. Dose response curve generated by treating ESCs (from 3 participants) with four doses of ASA (0.01mM, 0.1mM, 1mM, and 2.5mM) followed by stimulation with cAMP and measuring IGFBP1, a marker of decidualization. ESCs are from 2 control participants and 1 participant with endometriosis (triangle). More robust responses were observed with 1mM ASA and 2.5mM ASA doses; these doses were selected for future experiments. The red line is set at a fold change of 1 representing the response of vehicle (Veh)-treated ESCs.

Supplementary Figure 2A-H There was no difference in endometrial stromal cell (ESC) decidualization measured by IGFBP1 or PRL levels among participants regardless of disease state (control vs. endometriosis)

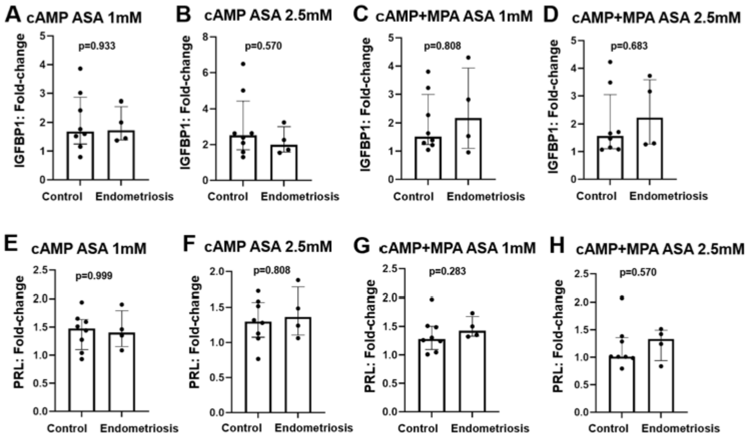

Supplementary Figure 2. Treatment of control- vs. endometriosis-ESCs with aspirin (ASA) (1-2.5mM) prior to stimulation with either (A, B) cAMP alone or (C, D) cAMP+MPA did not show statistically significant differences in enhanced decidualization markers, as determined by IGFBP1 levels by ELISA. Treatment of control- vs. endometriosis-ESCs with aspirin (ASA) (1-2.5mM) prior to stimulation with either (E, F) cAMP alone or (G, H) cAMP+MPA did not show statistically significant differences in enhanced decidualization markers, as determined by PRL levels by ELISA. There was no difference in endometrial stromal cell (ESC) decidualization markers measured by IGFBP1 (A-D) or PRL (E-H) among participants regardless of disease state (control vs. endometriosis). Each dot represents data from ESCs isolated from one participant. Mann-Whitney U test was used for statistical analysis; p-values are shown.

Supplementary Figure 3A-H ASA (2.5mM) enhances decidualization markers of ESCs

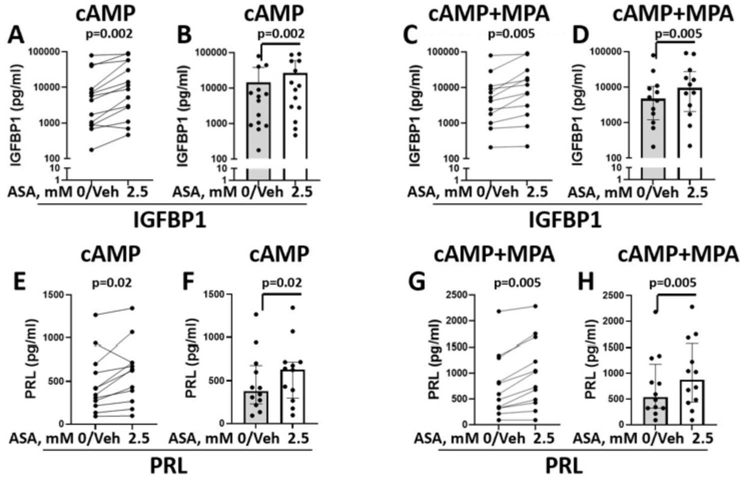

Supplementary Figure 3. Treatment of ESCs with ASA (2.5mM) prior to stimulation with either (A-B) cAMP alone or (C-D) cAMP+MPA enhances decidualization markers, as determined by raw IGFBP1 protein levels by ELISA when compared to vehicle (Veh)-treated ESCs. Treatment of ESCs with ASA (2.5mM) prior to stimulation with either (E-F) cAMP alone or (G-H) cAMP+MPA enhances decidualization markers, as determined by raw PRL protein levels by ELISA compared to vehicle. Raw IGFBP1 and PRL values are shown as paired data points (connected by a line) for each individual's ESCs (A, C: IGFBP1 and E, G: PRL). Raw IGFBP1 and PRL values are shown for each group (B and D: IGFBP1 and F and H: PRL); data are shown as median and interquartile range (IQR). Each dot represents data from ESCs isolated from one participant. Significance was determined by Wilcoxon signed-rank test for paired samples; p-values are shown.

Supplementary Figure 4A-D There was no difference in cytotoxicity among participants regardless of disease state (control vs. endometriosis)

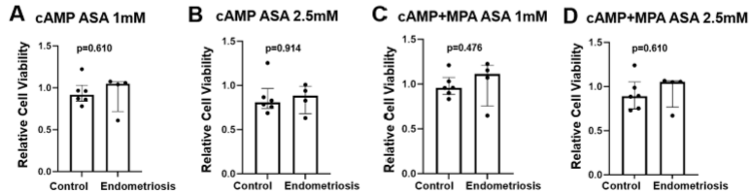

Supplementary Figure 4. Assessment of ESC cytotoxicity following treatment with aspirin (ASA) (1-2.5mM) prior to stimulation with either (A, B) cAMP alone or (C, D) cAMP+MPA did not reveal differences in cytotoxicity, as determined by Neutral Red uptake assay among ESCs obtained from participants based on disease state (control vs. endometriosis). Each dot represents data from ESCs isolated from one participant. The Mann-Whitney U test was used for statistical analysis; p-values are shown.

Supplementary Figure 5A-D ASA (2.5mM) is not cytotoxic to endometrial stromal cells (ESCs)

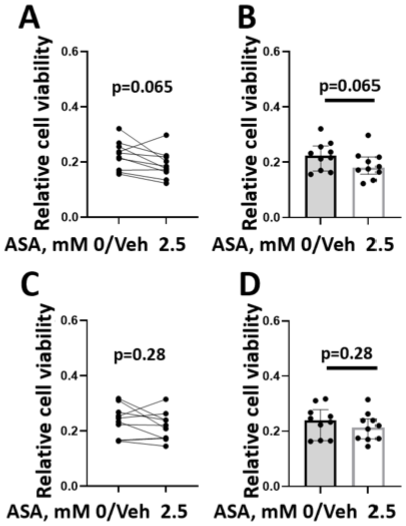

Supplementary Figure 5. Treatment of ESCs with ASA (2.5mM) prior to stimulation with either (A-B) cAMP alone or (C-D) cAMP+MPA was not cytotoxic, as determined by neutral red cellular assay when compared to vehicle (Veh)-treated ESCs. Raw relative cell viability values are shown as paired data points (connected by a line) for each individual's ESCs (A: cAMP, C: cAMP+MPA). Also, raw relative cell viability values are shown for each group (Veh vs. 2.5mM ASA) (B: cAMP, D cAMP+MPA); data are shown as median and interquartile range (IQR). Each dot represents raw data from ESCs isolated from one participant. Significance was determined by Wilcoxon signed-rank test for paired samples; p-values are shown.

Supplementary Figure 6A-B There was no difference in endometrial stromal cell (ESC) proliferation among participants regardless of disease state (control vs. endometriosis)

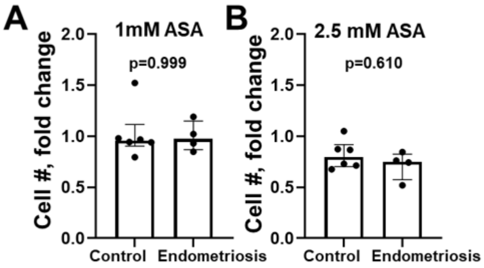

Supplementary Figure 6. Proliferation results did not differ among participants' ESCs regardless of disease state (control vs. endometriosis) at either aspirin (ASA) concentration studied (A) ASA (1mM) or (B) ASA (2.5mM). Each dot represents data from ESCs isolated from one participant. The Mann-Whitney U test was used for statistical analysis; p-values are shown.

Supplementary Figure 7A-B ASA (2.5mM) reduces endometrial stromal cell (ESC) proliferation

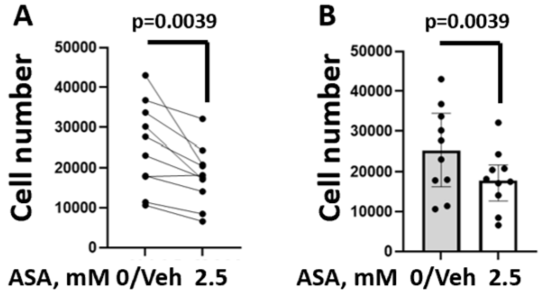

Supplementary Figure 7. ASA treatment (2.5mM) decreased proliferation of ESCs when compared to vehicle (Veh)-treated ESCs. Cell numbers are shown as paired data points (connected by a line) for each individual's ESCs (A). Cell numbers are shown for each group (Veh vs. 2.5mM ASA); data are shown as median and interquartile range (IQR) (B). Each dot represents data from ESCs isolated from one participant. Significance was determined by Wilcoxon signed-rank test for paired samples; p-values are shown
