# Supplementary material for: Aspirin enhances endometrial decidualization markers in vitro among women with and without endometriosis
Source: Reprod Fertil. 2026 Mar 26;7(1):RAF250034. doi: 10.1530/RAF-25-0034 (PMC13034528; doi:10.1530/RAF-25-0034)
Supplement: Supplementary file 2 [file supplementary_figure_8.pdf]

Uncropped Western Blots

Participant 1: 2/7/24

Participant 2: 6/17/25

Participants 3-5: 7/7/25

Participant 6:7/28/25

Participant #1

The differential effects of Aspirin (ASA) on AKT cell signaling pathway

pAKT

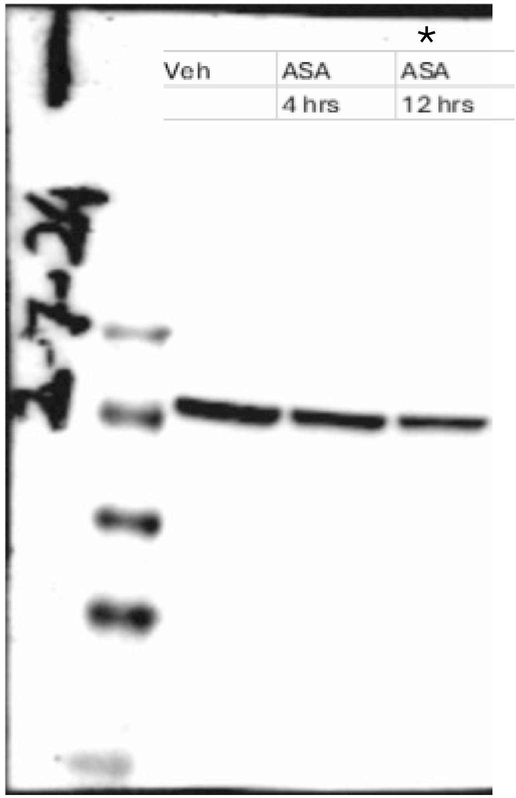

AKT

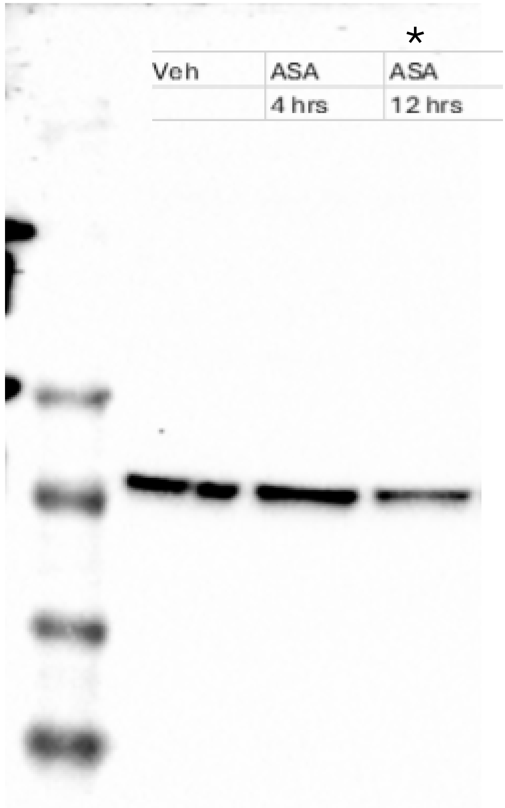

GAPDH

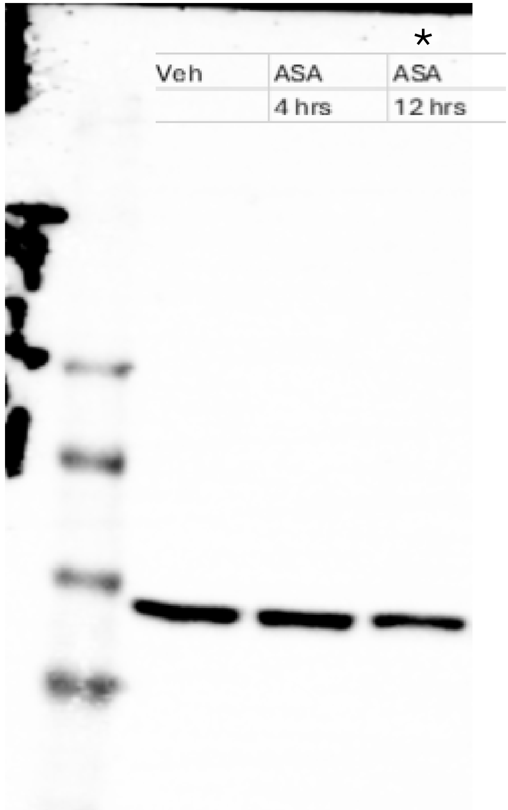

*Not included in manuscript

Participant #2

The differential effects of Aspirin (ASA) on cell signaling pathways

pAKT

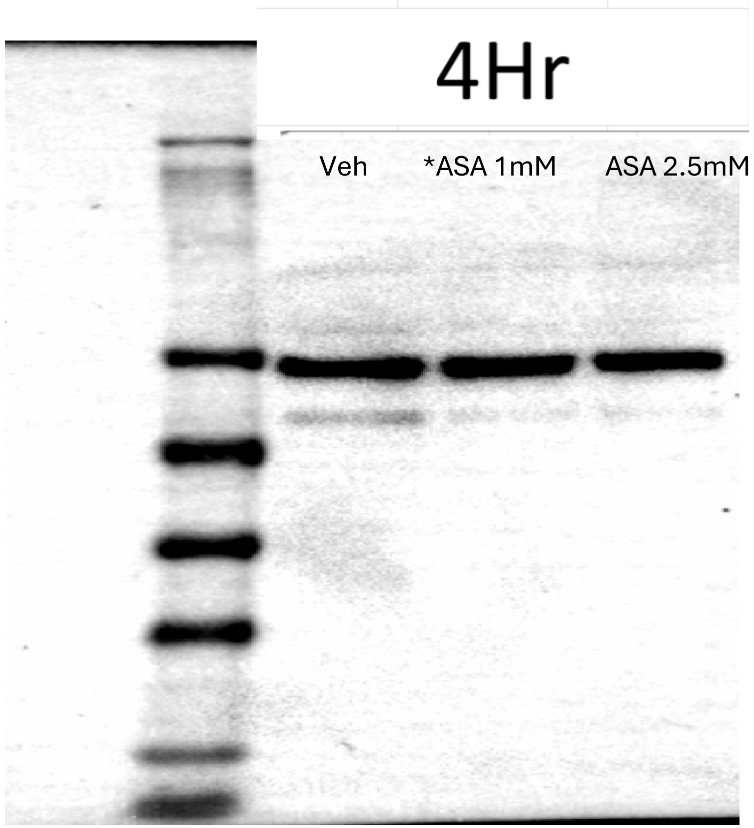

AKT

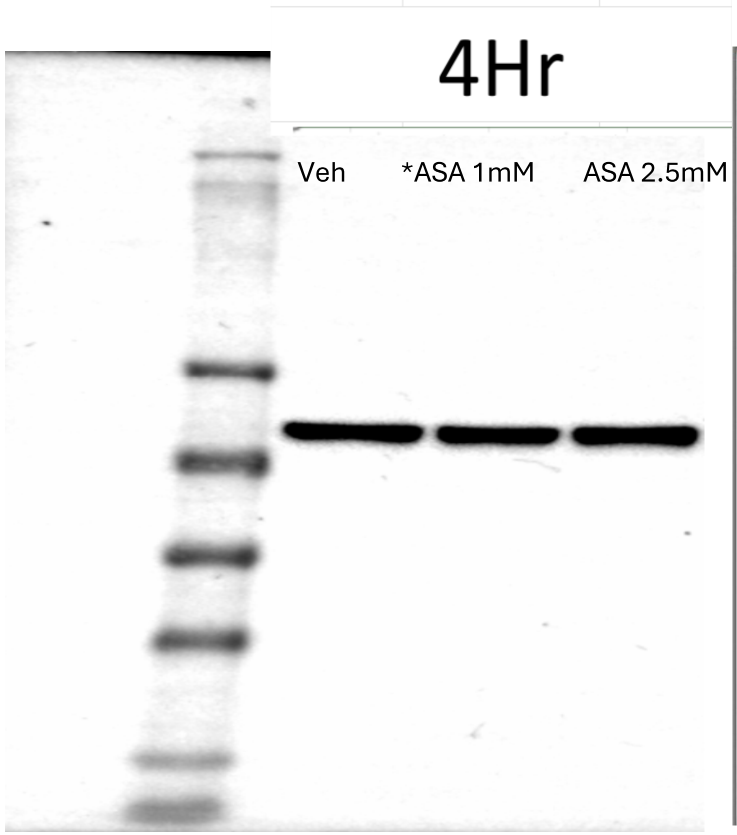

GAPDH

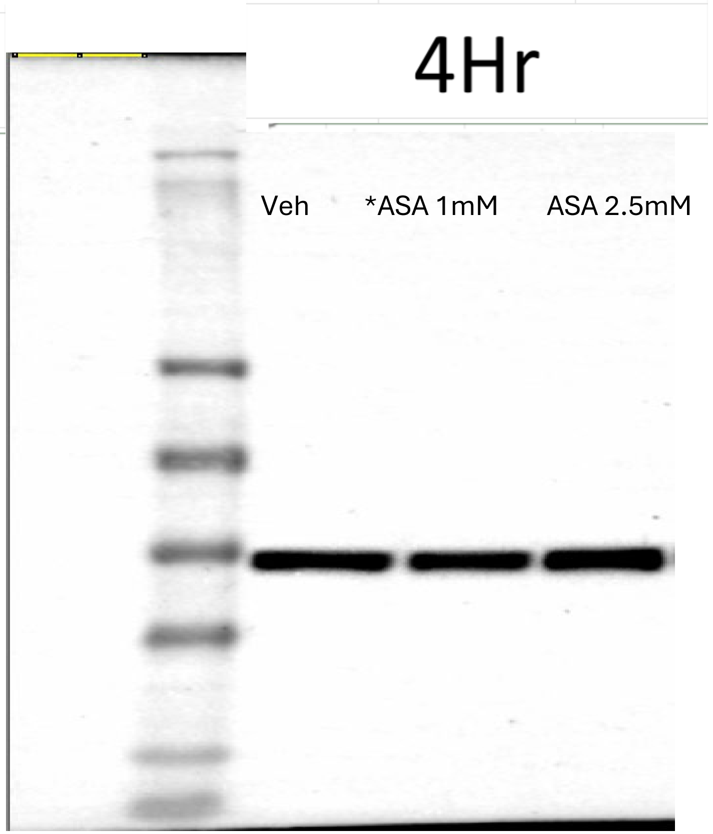

*Not included in manuscript

Participants #3-5

The differential effects of Aspirin (ASA) on cell signaling pathways

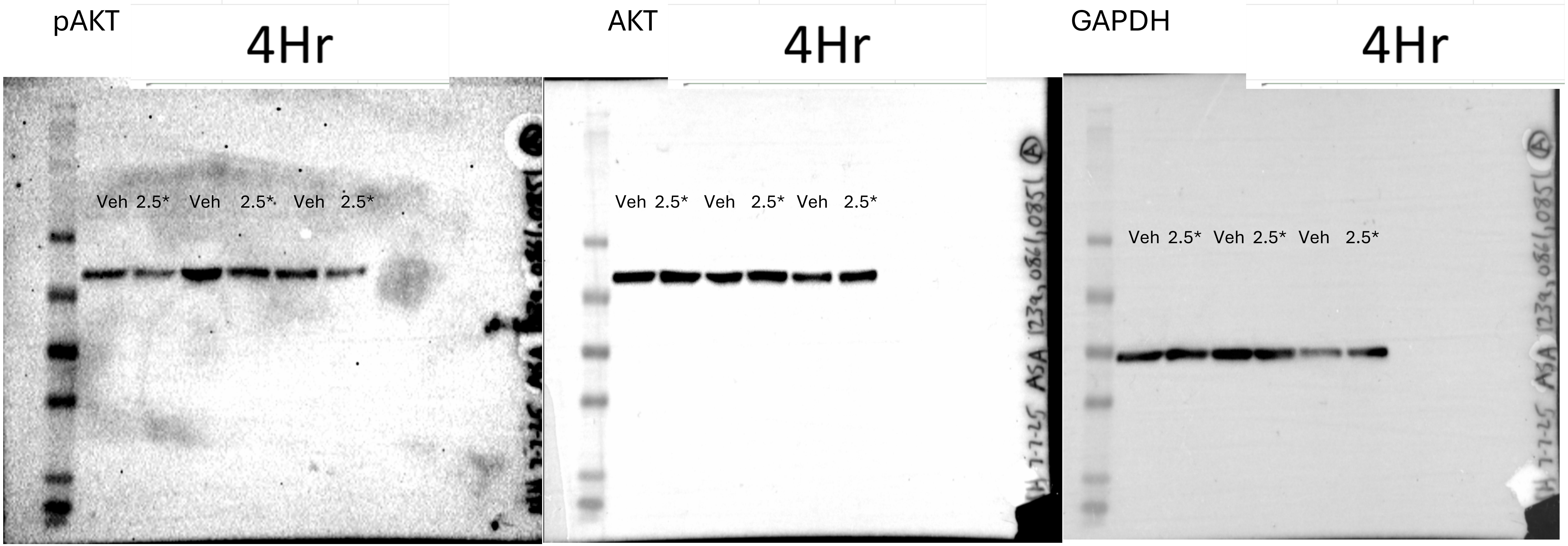

*Concentration given in mM

Participant #6

The differential effects of Aspirin (ASA) on cell signaling pathways

pAKT

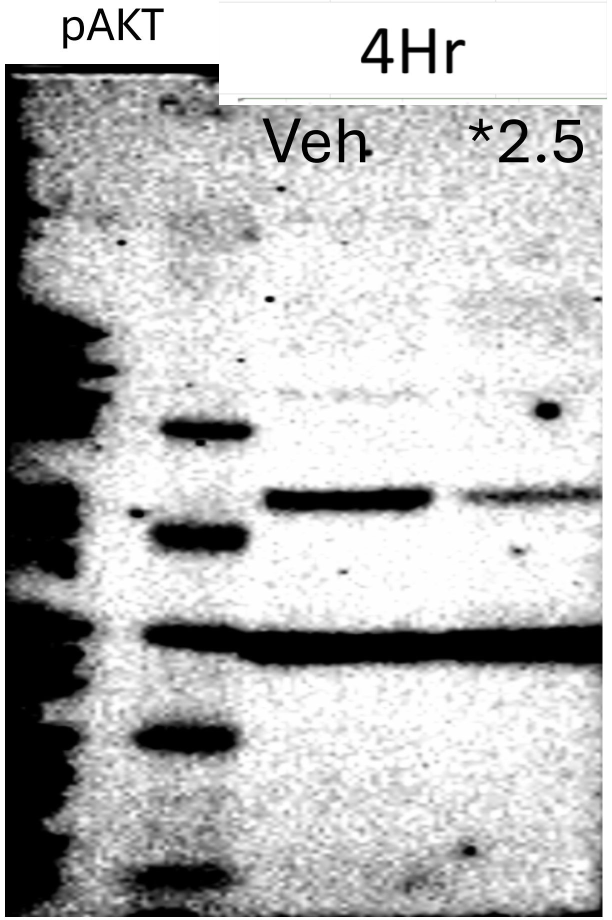

AKT

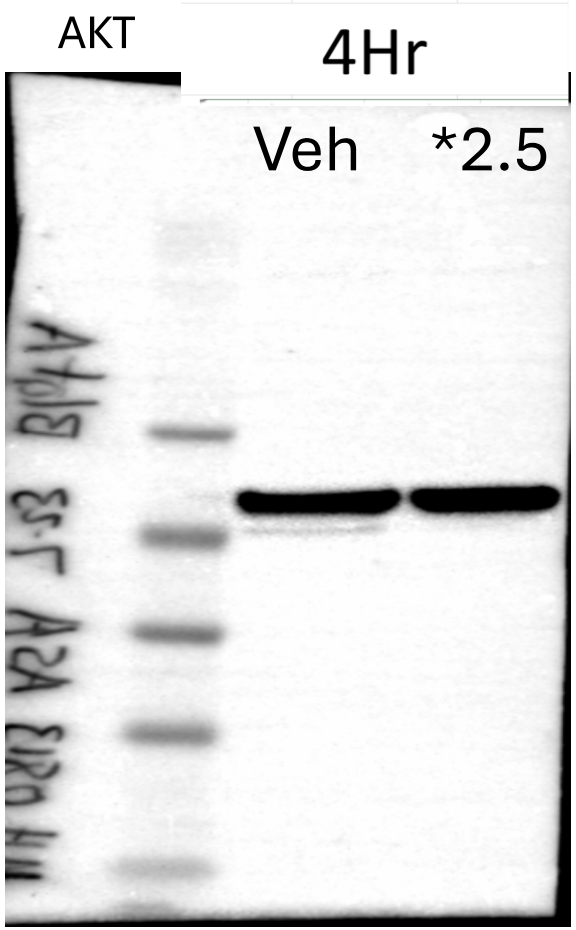

GAPD

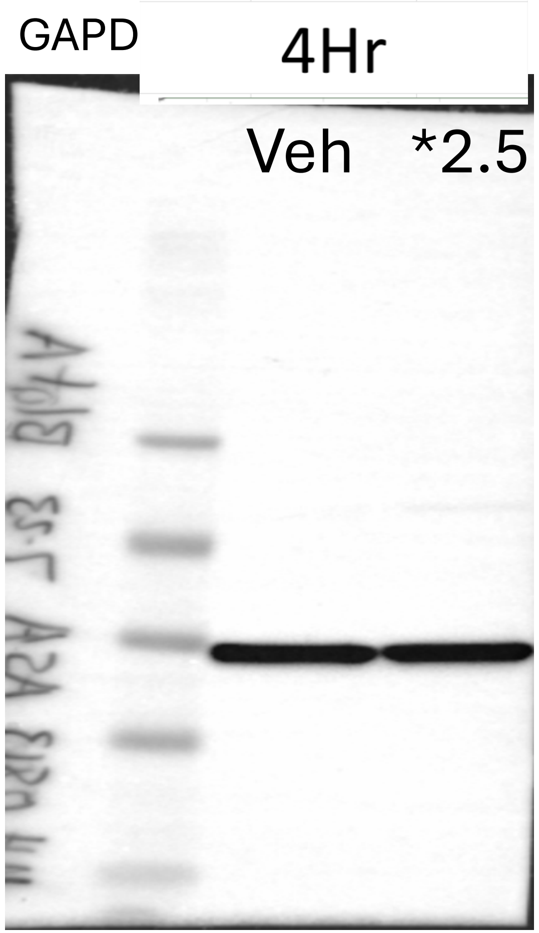

*Concentration given in mM

Band quantification

Participant 1: 2/7/24

	pAKT Density		AKT Density		GAPDH Density
Veh	89.70	Veh	87.29	Veh	84.12
2.5mM	66.59	2.5mM	81.17	2.5mM	76.02

Normalized	% Veh	pAKT/AKT	%Veh	Normalized	% Veh
1.07	100.00	1.03	100.00	1.04	100.00
0.88	82.14	0.82	79.82	1.07	102.90

Participant 2: 6/17/25

	pAKT Density		AKT Density		GAPDH Density
Veh	202.21	Veh	199.79	Veh	195.56
2.5mM	174.02	2.5mM	195.68	2.5mM	197.75

Normalized	% Veh	pAKT/AKT	%Veh	Normalized	% Veh
1.03	100.00	1.01	100.00	1.02	100.00
0.88	85.11	0.89	87.87	0.99	96.86

Participant 3-5: 7/7/25

	pAKT Density		AKT Density		GAPDH Density
Veh	142.76	Veh	186.45	Veh	150.53
2.5mM	104.96	2.5mM	187.00	2.5mM	161.25
Veh	179.29	Veh	153.25	Veh	180.74
2.5mM	153.07	2.5mM	178.99	2.5mM	151.28
Veh	149.22	Veh	133.25	Veh	76.42
2.5mM	81.66	2.5mM	157.29	2.5mM	122.16

Normalized	% Veh	pAKT/AKT	%Veh	Normalized	% Veh
0.95	100.00	0.77	100.00	1.24	100.00
0.65	68.63	0.56	73.30	1.16	93.63
0.99	100.00	1.17	100.00	0.85	100.00
1.01	102.00	0.86	73.10	1.18	139.54
1.95	100.00	1.12	100.00	1.74	100.00
0.67	34.24	0.52	46.36	1.29	73.85

Participant 6: 7/23/25

	pAKT Density		AKT Density		GAPDH Density
Veh	208.46	Veh	207.45	Veh	204.33
2.5mM	106.33	2.5mM	214.58	2.5mM	201.04

Normalized	% Veh	pAKT/AKT	%Veh	Normalized	% Veh
1.02	100.00	1.00	100.00	1.02	100.00
0.53	51.84	0.50	49.31	1.07	105.13

pAKT/AKT (%VEH)	VEH	2.5mM
Participant 1: 2/7/24	100	79.81981
Participant 2: 6/17/25	100	87.86627
Participant 3: 7/7/25	100	73.30265
Participant 4: 7/7/25	100	73.09838
Participant 5: 7/7/25	100	46.36054
Particiapant 6: 7/23/25	100	49.31011
