# Supplementary figures and images for: Aspirin enhances endometrial decidualization markers in vitro among women with and without endometriosis
Source: Reprod Fertil. 2026 Mar 26;7(1):RAF250034. doi: 10.1530/RAF-25-0034 (PMC13034528; doi:10.1530/RAF-25-0034)

## COX-1

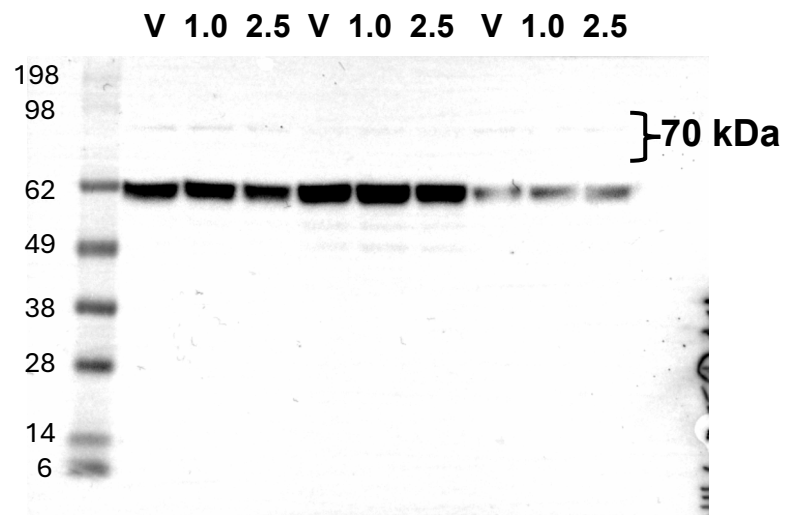

## COX-1

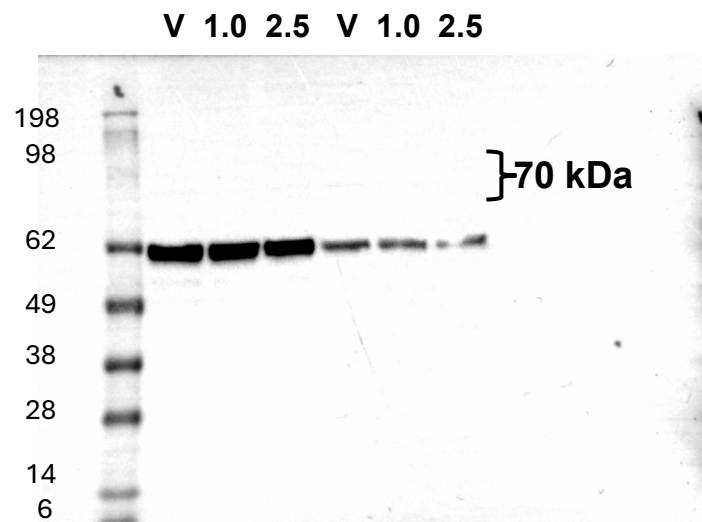

## COX-1

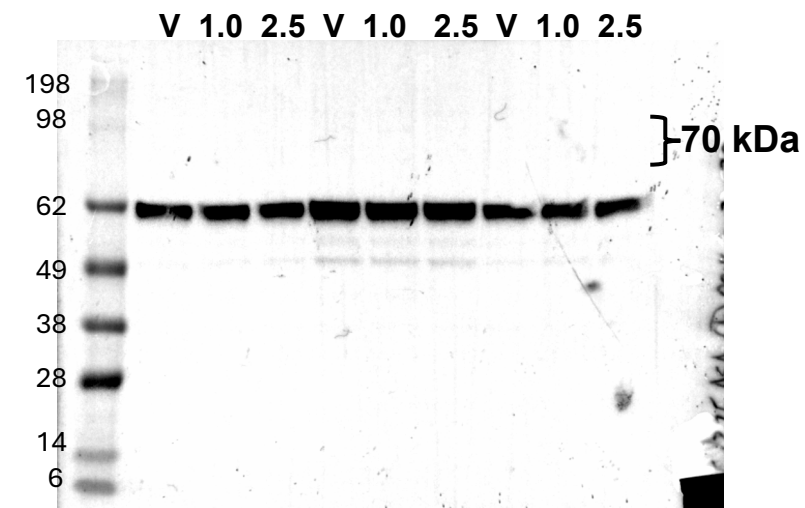

## COX-2

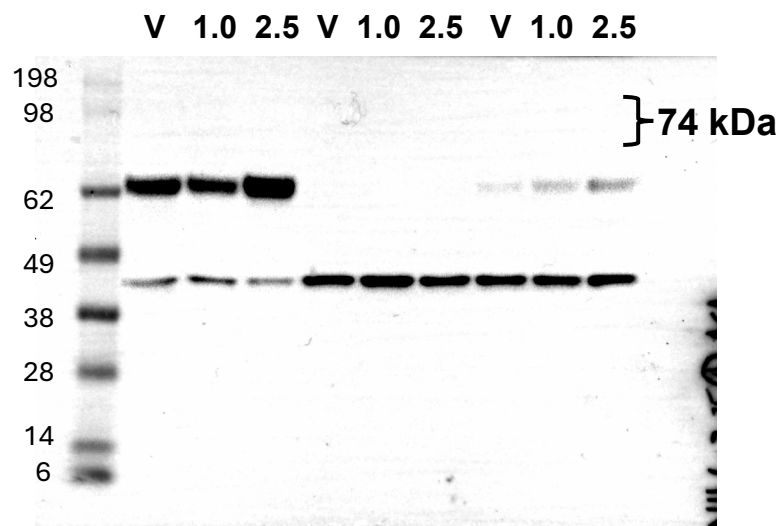

## COX-2

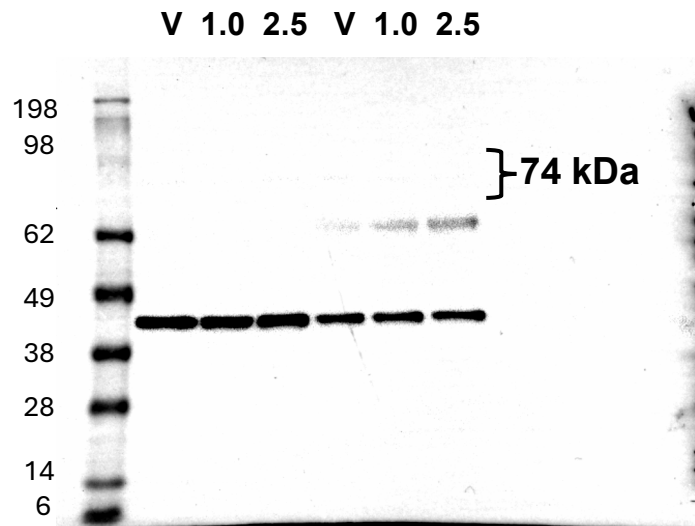

## COX-2

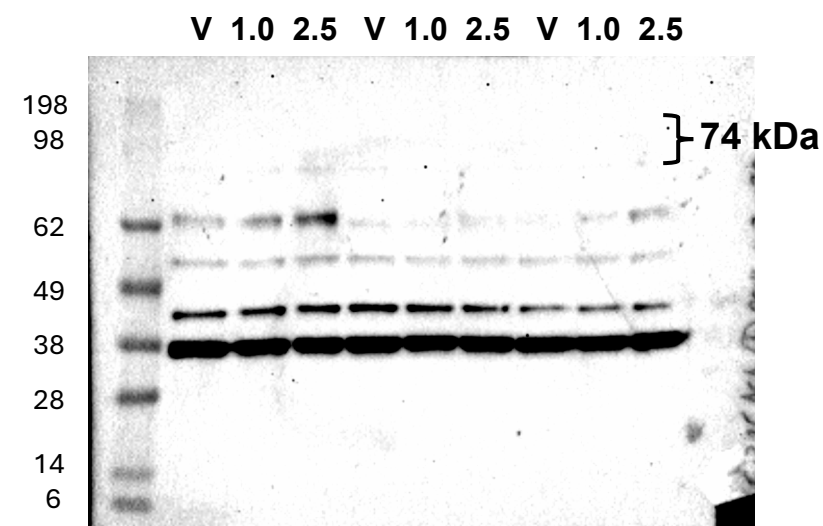

Supplement: Supplementary file 3 [file supplementary_figure_9.pdf]
